# Multi-trait phenotyping under reproductive-stage heat stress reveals heritable reproductive and yield indicators of tolerance in common bean (*Phaseolus vulgaris* L.)

**DOI:** 10.3389/fpls.2026.1904240

**Published:** 2026-08-11

**Authors:** Aastha Sharda, Shikha Chaudhary, Bhawna Kumari, Mohar Singh, Uday C. Jha, Kamal Dev Sharma, P. V. Vara Prasad, Kadambot H. M. Siddique, Harsh Nayyar

**Affiliations:** 1 Department of Botany, Panjab University, Chandigarh, India; 2 Indian Council of Agricultural Research (ICAR)- National Bureau of Plant Genetic Resources Regional Station, Shimla, Himachal Pradesh, India; 3 Crop Improvement Division, Indian Institute of Pulses Research, Kanpur, Uttar Pradesh, India; 4 Department of Agricultural Biotechnology, Chaudhary Sarwan Kumar (CSK) Himachal Pradesh Agricultural University, Palampur, Himachal Pradesh, India; 5 Department of Agronomy, Kansas State University, Manhattan, KS, United States; 6 The UWA Institute of Agriculture, The University of Western Australia, Perth, WA, Australia

**Keywords:** common bean accessions, heat stress, *Phaseolus vulgaris* L., physiological traits, pollen germination, reproductive thermotolerance, stigma receptivity, yield stability

## Abstract

Common bean (*Phaseolus vulgaris* L.) exhibits significant susceptibility to heat stress during its reproductive phase. However, the physiological and reproductive traits underlying heat tolerance remain inadequately characterised across diverse bean germplasm. In this study, 56 accessions were assessed under controlled heat stress conditions (32/22 °C day/night) and optimal conditions (25/15 °C) during the flowering-to-podding stage over a seven-day period across two consecutive growing seasons (2023 and 2024). Seventeen traits encompassing phenological, growth, physiological, reproductive, and yield categories were evaluated. Heat stress resulted in severe and consistent yield reductions across both seasons, with the extent of these reductions varying significantly between the tolerant and sensitive accessions. Single seed weight decreased by approximately 8% in tolerant accessions compared to approximately 34% in sensitive accessions, whereas total seed weight decreased by approximately 50% and 71%, respectively, with differences that were highly reproducible across both growing seasons. Pollen viability, pollen germination, stigma receptivity, and ovule viability were the most pronounced differentiators between the groups. Pollen viability decreased by 55–59% in tolerant accessions compared to 64–72% in sensitive lines, and pollen germination decreased by 59–65% and 71–79% across both years, respectively. In addition, tolerant accessions exhibited substantially lower electrolyte leakage and smaller declines in stomatal conductance, chlorophyll content, and photosynthetic efficiency. Two-way ANOVA revealed significant effects of accession, treatment, and their interaction for the key reproductive and yield traits. Regression analysis identified total seed weight ratio (R² = 0.876 and 0.801) and single seed weight ratio (R² = 0.522 and 0.523) as the traits most strongly associated with heat tolerance, whilst pollen viability (R² = 0.449 and 0.488), pollen germination (R² = 0.428 and 0.460), ovule viability (R² = 0.438 and 0.387), and stigma receptivity (R² = 0.428 and 0.366) showed moderate associations. Cluster and principal component analyses consistently identified IC-14912, EC-106613, IC-545783, and IC-16906 as heat-tolerant and IC-545792, EC-100098, EC-121013, and IC-362075 as heat-sensitive in both seasons. The identified heat-tolerant accessions represent priority genetic resources for heat tolerance breeding programmes in common bean.

## 1 Introduction

The common bean (*Phaseolus vulgaris* L.) is one of the most nutritionally significant grain legumes globally, serving as a primary source of dietary protein, essential vitamins, and minerals for millions of individuals, particularly in the developing regions of Latin America, sub-Saharan Africa, and South Asia (Mukankusi et al., 2019; Soltani et al., 2021; Uebersax et al., 2023; Asrat and Abera, 2025). In 2022, the global production of dry common bean reached approximately 28 million tonnes, with India and Myanmar being the leading producers (FAOSTAT, 2024). Despite their substantial nutritional and economic importance, the cultivation of common bean is severely constrained by various abiotic stresses, with heat, drought, and soil salinity being the most frequently reported (Beebe et al., 2014).

Amongst the various determinants of productivity, elevated temperatures are a significant constraint. The crop under consideration is adapted to temperate climates and demonstrates inherent sensitivity to high temperatures, with yields declining markedly when daytime temperatures exceed 30 °C or nighttime temperatures remain above 20 °C. These thresholds are increasingly being surpassed in major agricultural regions (Soltani et al., 2019; Suárez et al., 2020; Mahajan et al., 2023). The reproductive growth stage is particularly vulnerable, as heat stress during this phase precipitates the convergence of failures across multiple plant processes (Ahmed et al., 1992; Ismail and Hall, 1999; Omae et al., 2012; Kaushal et al., 2013). These failures encompass reduced pollen viability, anther indehiscence, impaired pollen tube growth, and disruption of ovule development, collectively resulting in flower and bud abortion, poor seed filling, defective seed formation, and a high incidence of parthenocarpic pods, ultimately leading to substantial yield losses (Gross and Kigel, 1994; Silva et al., 2019; Rose et al., 2023; Mahajan et al., 2023). Substantial yield reductions have been well documented in common bean and related legumes under field heat stress conditions, with losses ranging from 26% to 37%, where elevated nighttime temperatures often prove more detrimental than peak daytime temperatures (Barros et al., 2021; Vargas et al., 2021). Considerable effort has been directed towards identifying heat-tolerant germplasm and elucidating the mechanisms of tolerance. A large-scale evaluation of 196 accessions from five *Phaseolus* species confirmed that most accessions of *P. acutifolius*, *P. lunatus*, and *P. coccineus* exhibit greater resistance to heat stress than the common bean (Rainey and Griffiths, 2005; Tene et al., 2025). In a parallel approach, the development of an interspecific population using a bridging accession to introgress traits from wild tepary bean led to the identification of 27 significant quantitative trait loci (QTLs) associated with yield under high temperatures (Cruz et al., 2023). At the trait level, the reproductive sensitivity of common bean to heat stress is sharply illustrated by data showing that pollen viability in the heat-sensitive cultivar ‘Calima’ declined to 24.9% under 31/24 °C conditions, whereas a tolerant breeding line (HTA4) retained 71.1% viability under identical stress (Rose et al., 2023). Despite these advances, many investigations have primarily relied on field-based phenological screening or QTL-based analyses (Blair et al., 2006; Porch et al., 2013; Díaz et al., 2020), and comprehensive trait-level evaluations of diverse common bean accessions under controlled heat stress, integrating phenological, growth, physiological, reproductive, and yield responses simultaneously are limited (Basu et al., 2019; Jiang et al., 2020; Chaudhary et al., 2022; Biradar et al., 2025). Therefore, assessing existing common bean germplasm through an integrated multi-trait approach to identify heat-tolerant accessions and characterising their underlying physiological and reproductive mechanisms remains a priority (López-Hernández and Cortés, 2019; Oladzad et al., 2019; Bomers et al., 2022).

This study aimed to address this gap by evaluating 56 common bean accessions under controlled heat stress conditions (32/22 °C day/night) during the reproductive stage. Physiological traits, including photosynthetic efficiency, membrane stability, stomatal conductance, and chlorophyll retention, were assessed alongside yield components, reproductive traits, and phenological characteristics to develop comprehensive trait profiles across contrasting accessions. Pollen viability, pollen germination, stigma receptivity, and ovule viability were examined as indicators of reproductive performance under stress, given their direct influence on fertilisation efficiency and seed set. This integrated approach, applied across two consecutive growing seasons, was designed to identify heat-tolerant accessions, establish trait-based indicators of heat tolerance, and provide physiological and agronomic criteria applicable to breeding programmes aimed at developing heat-resilient common bean cultivars suited to increasing temperature environments.

## 2 Materials and methods

### 2.1 Experimental setup and growth conditions

**Figure 1:**
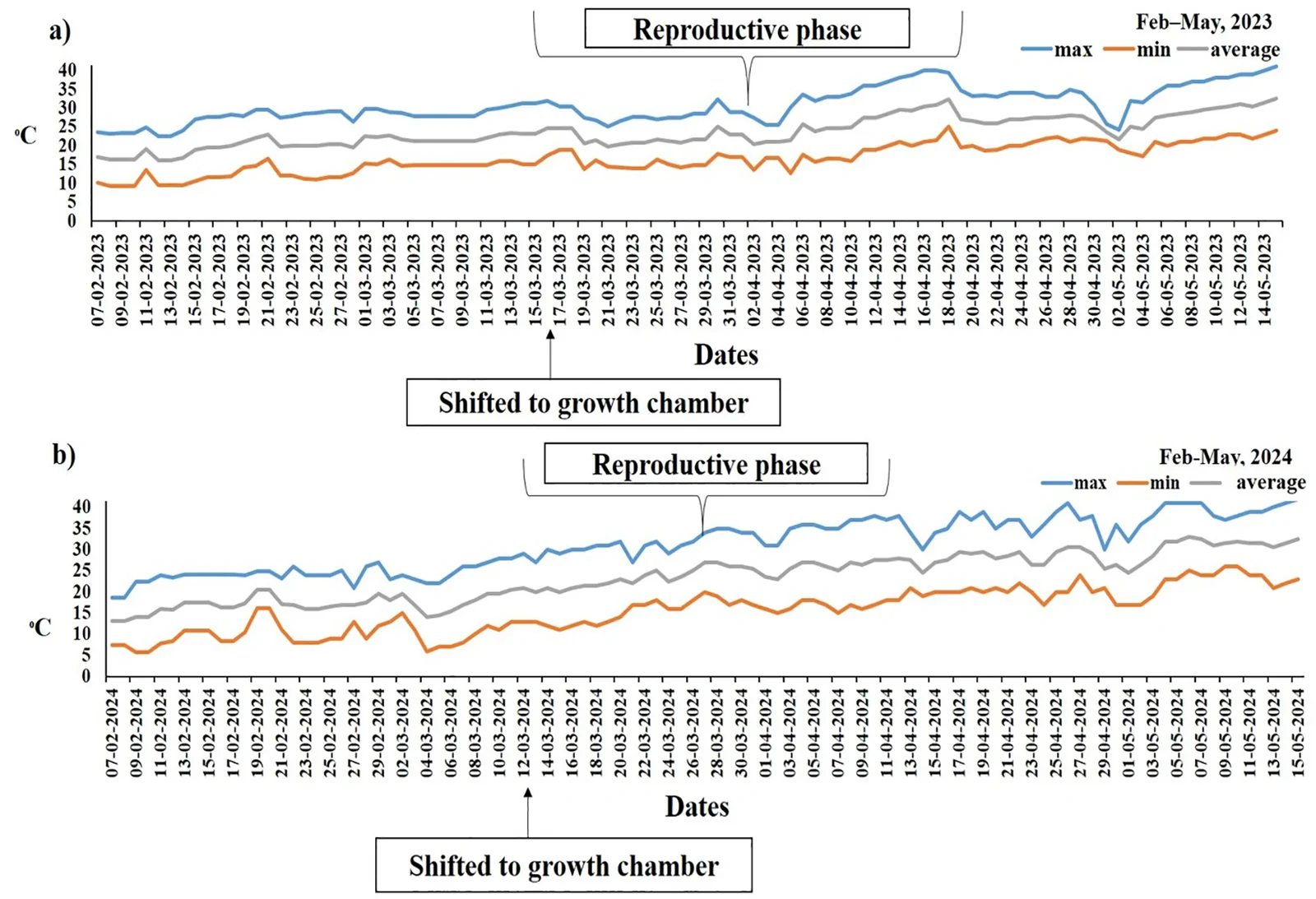
Temperature profile (maximum, minimum, and average) recorded for plants grown under outdoor conditions across two consecutive growing seasons: **(a)** 2023 and **(b)** 2024, during heat stress screening of common bean accessions.

Fifty-six accessions of common bean (*Phaseolus vulgaris* L.) (**Table 1**) were obtained from the National Bureau of Plant Genetic Resources (NBPGR), Regional Station, Phagli, Shimla, Himachal Pradesh, India. These accessions were assessed for their responses to heat stress across phenological, growth, physiological, reproductive, and yield-related traits during two consecutive growing seasons. The experiments were conducted during the summer seasons of 2023 and 2024 at the Department of Botany, Panjab University, Chandigarh, India (30.75° N, 76.78° E). Sowing was performed on 7 February in both years, a period characterised by progressively increasing day and night temperatures in Chandigarh, ensuring that the crop entered its reproductive phase under naturally escalating thermal conditions. Prior to sowing, the seeds were inoculated with *Rhizobium leguminosarum* bv. *phaseoli* at a rate of 1.95 g kg^-^¹ seed to facilitate effective nodulation. The plants were cultivated in 4-kg pots containing a mixture of sandy loam soil and sand (3:1), enriched with farmyard manure at a soil-to-manure ratio of 3:1. The potting medium was also supplemented with fertilizers prior to sowing to ensure adequate nutrient availability throughout plant growth. An initial basal application of urea, Single Super Phosphate (SSP), and Muriate of Potash (MOP) (0.5 g each dissolved in 2 L of water) was uniformly applied to all pots before sowing. Subsequently, two additional fertilizer applications were made at 2–3 and 4–6 weeks after sowing. During each application, urea (0.5 g), Single Super Phosphate (SSP) (0.25 g), and Muriate of Potash (MOP) (0.25 g) were dissolved in 2 L of water and applied uniformly to all pots. The second fertilizer application was carried out under outdoor conditions before the plants were transferred to the walk-in growth chamber, whereas the third fertilizer application was applied inside the growth chamber after plant transfer. The fertilizer concentrations described above represent the standard formulation per 2 L of water. Larger volumes of the nutrient solution were prepared as required whilst maintaining the same fertilizer concentrations to ensure uniform application across all experimental pots. The same fertilisation schedule was maintained across all treatments and in both experimental years to ensure uniform nutrient availability and eliminate nutritional variation as a confounding factor in assessing the effects of heat stress. Five seeds were sown per pot and thinned to three healthy, uniform plants after germination to maintain a consistent plant density. Maximum and minimum temperatures and relative humidity were monitored continuously from sowing until the flowering stage (**Figure 1**).

**Table 1:** List of 56 common bean accessions used in the present study.

S. no.	Accession	Source
1.	EC-100098	ICAR-NBPGR*, Shimla, Himachal Pradesh, India
2.	EC-100678	ICAR-NBPGR, Shimla, Himachal Pradesh, India
3.	EC-103346	ICAR-NBPGR, Shimla, Himachal Pradesh, India
4.	EC-106613	ICAR-NBPGR, Shimla, Himachal Pradesh, India
5.	EC-109731	ICAR-NBPGR, Shimla, Himachal Pradesh, India
6.	EC-115962	ICAR-NBPGR, Shimla, Himachal Pradesh, India
7.	EC-116177	ICAR-NBPGR, Shimla, Himachal Pradesh, India
8.	EC-121013	ICAR-NBPGR, Shimla, Himachal Pradesh, India
9.	EC-125715	ICAR-NBPGR, Shimla, Himachal Pradesh, India
10.	EC-127580	ICAR-NBPGR, Shimla, Himachal Pradesh, India
11.	EC-127644	ICAR-NBPGR, Shimla, Himachal Pradesh, India
12.	EC-127645	ICAR-NBPGR, Shimla, Himachal Pradesh, India
13.	EC-128277	ICAR-NBPGR, Shimla, Himachal Pradesh, India
14.	EC-129372	ICAR-NBPGR, Shimla, Himachal Pradesh, India
15.	EC-241420	ICAR-NBPGR, Shimla, Himachal Pradesh, India
16.	EC-241422	ICAR-NBPGR, Shimla, Himachal Pradesh, India
17.	EC-241425	ICAR-NBPGR, Shimla, Himachal Pradesh, India
18.	EC-253417	ICAR-NBPGR, Shimla, Himachal Pradesh, India
19.	EC-271483	ICAR-NBPGR, Shimla, Himachal Pradesh, India
20.	EC-271488	ICAR-NBPGR, Shimla, Himachal Pradesh, India
21.	EC-271492	ICAR-NBPGR, Shimla, Himachal Pradesh, India
22.	EC-271499	ICAR-NBPGR, Shimla, Himachal Pradesh, India
23.	EC-271500	ICAR-NBPGR, Shimla, Himachal Pradesh, India
24.	EC-271501	ICAR-NBPGR, Shimla, Himachal Pradesh, India
25.	EC-271505	ICAR-NBPGR, Shimla, Himachal Pradesh, India
26.	IC-10051	ICAR-NBPGR, Shimla, Himachal Pradesh, India
27.	IC-14711	ICAR-NBPGR, Shimla, Himachal Pradesh, India
28.	IC-14912	ICAR-NBPGR, Shimla, Himachal Pradesh, India
29.	IC-16906	ICAR-NBPGR, Shimla, Himachal Pradesh, India
30.	IC-47655	ICAR-NBPGR, Shimla, Himachal Pradesh, India
31.	IC-47656	ICAR-NBPGR, Shimla, Himachal Pradesh, India
32.	IC-361905	ICAR-NBPGR, Shimla, Himachal Pradesh, India
33.	IC-361916	ICAR-NBPGR, Shimla, Himachal Pradesh, India
34.	IC-362075	ICAR-NBPGR, Shimla, Himachal Pradesh, India
35.	IC-362079	ICAR-NBPGR, Shimla, Himachal Pradesh, India
36.	IC-362082	ICAR-NBPGR, Shimla, Himachal Pradesh, India
37.	IC-545783	ICAR-NBPGR, Shimla, Himachal Pradesh, India
38.	IC-545790	ICAR-NBPGR, Shimla, Himachal Pradesh, India
39.	IC-545791	ICAR-NBPGR, Shimla, Himachal Pradesh, India
40.	IC-545792	ICAR-NBPGR, Shimla, Himachal Pradesh, India
41.	IC-547540	ICAR-NBPGR, Shimla, Himachal Pradesh, India
42.	IC-556437	ICAR-NBPGR, Shimla, Himachal Pradesh, India
43.	IC-556442	ICAR-NBPGR, Shimla, Himachal Pradesh, India
44.	IC-556443	ICAR-NBPGR, Shimla, Himachal Pradesh, India
45.	IC-556455	ICAR-NBPGR, Shimla, Himachal Pradesh, India
46.	IC-556457	ICAR-NBPGR, Shimla, Himachal Pradesh, India
47.	IC-556461	ICAR-NBPGR, Shimla, Himachal Pradesh, India
48.	IC-556464	ICAR-NBPGR, Shimla, Himachal Pradesh, India
49.	IC-568172	ICAR-NBPGR, Shimla, Himachal Pradesh, India
50.	IC-568183	ICAR-NBPGR, Shimla, Himachal Pradesh, India
51.	Jawala (Released varieties)	CSKHPKV**, Palampur Himachal Pradesh, India
52.	Baspa (Released varieties)	CSKHPKV, Palampur Himachal Pradesh, India
53.	Triloki (Released varieties)	CSKHPKV, Palampur Himachal Pradesh, India
54.	Kailash (Released varieties)	CSKHPKV, Palampur Himachal Pradesh, India
55.	PLB10-1 (Promising selection)	ICAR-NBPGR, Shimla, Himachal Pradesh, India
56.	PLB14-1 (Promising selection)	ICAR-NBPGR, Shimla, Himachal Pradesh, India

*ICAR-NBPGR, Indian Council of Agricultural Research–National Bureau of Plant Genetic Resources, Shimla, Himachal Pradesh, India.

**CSKHPKV, Chaudhary Sarwan Kumar Himachal Pradesh Krishi Vishvavidyalaya, Palampur, Himachal Pradesh, India.

Upon bud initiation, which exhibited slight variation amongst accessions due to differences in phenological development, the plants were relocated to a walk-in growth chamber for temperature treatment. The chamber temperature was incrementally increased by 1 °C per day until reaching the target of 32/22 °C (day/night), after which these conditions were maintained for seven days during the critical flowering-to-podding period of the plants. The chamber was set to a 12-hour photoperiod, with a light intensity of 600 µmol m^-^² s^-^¹ and a relative humidity of 65–70%. Concurrently, a non-stressed control was maintained for all 56 accessions at 25/15 °C (day/night) under an identical photoperiod, light intensity and humidity regime.

The selection of a seven-day duration for stress exposure was based on previous findings, which suggested that the effects of heat stress on reproductive and physiological traits typically manifest within five to seven days of temperature exposure. This timeframe aligns with critical reproductive processes in common bean, such as pollen development, stigma receptivity, and pod formation, and occurs prior to any potential recovery responses under moderate heat stress conditions (Gross and Kigel, 1994; Porch and Jahn, 2001). Upon reaching physiological maturity, the plants were harvested and assessed for the number of filled pods, seed count, and total seed weight per plant (TSW).

### 2.2 Trait measurement

All 56 accessions were assessed for various phenological, growth, physiological, reproductive, and yield-related traits. Growth- and yield-related traits were documented at physiological maturity under both control and heat stress conditions, whereas physiological and reproductive parameters were evaluated immediately after the seven-day treatment period. To ensure comparability across accessions, heat stress was applied at the same reproductive stage for all accessions, and uniform growth chamber conditions were maintained throughout the treatment.

A total of 17 traits were recorded and categorised into five groups:

- Phenological traits included days to podding (DP) and days to maturity (DM).
- Growth traits included plant height (H), total biomass (BM), and harvest index (HI).
- Yield traits included the number of filled pods per plant (FP), number of seeds per plant (NS), single seed weight (SSW), and total seed weight per plant (TSW).
- Physiological traits included membrane stability assessed through electrolyte leakage (EL), photosynthetic efficiency (PE; Fv/Fm ratio), chlorophyll content measured using SPAD values, and stomatal conductance (SC).
- Reproductive traits included pollen viability (PV), pollen germination (PG), stigma receptivity (SR), and ovule viability (OV).

### 2.3 Predicted yield (Ŷs) and heat tolerance indices

To assess heat tolerance independently of a genotype’s inherent yield potential, thereby circumventing the confounding effect of ranking accessions solely by yield magnitude, the regression-residual method of (Porch, 2006) was used. Seed yield under heat stress (Ys) was regressed against seed yield under control conditions (Yc) separately for each experimental year using linear regression analysis. Based on the derived regression equations between Yc and Ys, the expected seed yield under heat stress (Ŷs) was estimated. The regression slope (a) and intercept (b) values were 0.441 and −0.573 during the first year, whereas corresponding values for the second year were 0.438 and −0.581, respectively. Heat tolerance residuals (R) were calculated as the difference between the observed heat-stress yield and the predicted yield, expressed as R = Ys – Ŷs. Positive residuals indicate accessions that exceed expectations under stress, independent of the baseline yield level (Vadez et al., 2007).

The calculated residuals were subsequently employed as dependent variables in regression analyses against the following explanatory trait ratios (heat stress relative to control): i) seed yield ratio (Ys/Yc); ii) total biomass ratio; iii) seed number per plant ratio; iv) single seed weight ratio; v) harvest index ratio; vi) plant height ratio; vii) days to flowering; viii) days to podding ratio; and ix) days to maturity ratio. Days to flowering was additionally recorded and incorporated into the regression framework, although it was not part of the formal replicated trait evaluation process. Where appropriate, second-order polynomial (Type II) regression models were applied to more accurately describe the non-linear relationships between the heat tolerance residuals and the explanatory variables.

### 2.4 Physiological traits

Physiological parameters were assessed on fully expanded leaves of plants during the active treatment phase, following established methodologies (Heath and Packer, 1968; Siddiqui et al., 2015; Tiwari et al., 2018; Vargas et al., 2021; Jha et al., 2025). Membrane stability was quantified by evaluating electrolyte leakage (EL) through measurements of electrical conductivity before and after heat treatment (Thiaw and Hall, 2004). Stomatal conductance (SC) was measured using an SC-1 portable leaf porometer (Decagon Devices, Pullman, WA, USA), with measurements taken at approximately 11:00 h from fully expanded leaves at the second or third node below the shoot apex (Devi et al., 2022). Leaf chlorophyll content was estimated from SPAD readings obtained using a SPAD chlorophyll metre (Apogee Instruments, Logan, UT, USA) from the marked leaves between 10:00 and 11:00 h. Photosynthetic efficiency (PE; Fv/Fm ratio) was determined at 11:00 h using a modulated fluorometer (Model OS1-FL, Opti-Sciences, Tyngsboro, MA, USA) following the dark adaptation of the leaves, as described by Seidel et al. (2016).

### 2.5 Reproductive traits

Reproductive characteristics were assessed according to established methodologies (Silva et al., 2019; Vargas et al., 2021). Floral samples were obtained from plants maintained in a controlled growth chamber. Stigma and ovule samples were collected one day prior to anthesis, and pollen was sampled at anthesis. Pollen viability was determined by staining with 0.5% acetocarmine solution. Approximately 200 pollen grains per sample were examined microscopically, and grains exhibiting uniform morphology and intense red staining were classified as viable (Rose et al., 2023). Pollen germination was performed using Brewbaker and Kwack method. Germination was defined as the proportion of grains that produced elongated pollen tubes (Brewbaker and Kwack, 1963; Porch and Jahn, 2001). Stigma receptivity was evaluated using an esterase-based chromogenic assay with α-naphthaleneacetic acid and fast blue B salt, and the staining intensity was scored on a 1–5 scale (Mattsson et al., 1974). Ovule viability was assessed using the triphenyl tetrazolium chloride (TTC) reduction test, with the intensity of red pigmentation scored on a 1–5 scale (Traub et al., 2018).

### 2.6 Identification of heat-tolerant and heat-sensitive accessions

All 56 accessions were categorised into four response groups: heat tolerant (HT), moderately tolerant, moderately sensitive, and heat sensitive (HS), based on their composite performance across all measured traits over both experimental years. Accessions that consistently exhibited superior yield attributes, enhanced membrane stability, chlorophyll content, and photosynthetic efficiency, along with improved pollen viability, pollen germination, stigma receptivity, and ovule viability under heat stress conditions were classified as heat tolerant. In contrast, accessions that demonstrated the most pronounced reductions in these parameters were classified as heat-sensitive (Sharma et al., 2016). From these two extreme categories, four representative HT and HS accessions were selected for detailed comparative analyses. For each accession, three samples per replicate were collected, averaged, and used for subsequent calculations (Vargas et al., 2021).

### 2.7 Experimental design and statistical analysis

The experiment was implemented using a randomised complete block design (RCBD) with three replicates per treatment, assessed under both outdoor pot conditions and controlled walk-in growth chamber conditions. For physiological observations, one plant per replicate was utilised (three plants per accession per treatment), whereas yield traits were recorded from three plants per replicate (nine plants per accession per treatment). Data were analysed using two-way analysis of variance (ANOVA) in RStudio (stats package, version 4.3.0; R Core Team, 2023), with accession, treatment, replication, and accession × treatment interaction serving as sources of variation. The evaluated accessions were considered as genotypes for statistical analyses. The assumptions of ANOVA were verified using Q–Q plots and the gvlma package, and Tukey’s honestly significant difference test (p< 0.05) was used for mean comparisons. Genotypic variance (VG), error variance (VE), and phenotypic variance (VP) were estimated from ANOVA mean squares separately for each year under heat stress conditions. Variance components were estimated using expected mean squares from the RCBD ANOVA model. Broad-sense heritability (H²) under heat stress conditions was estimated using genotypic and error variance components according to the following formula:


$$H^{2}=\sigma^{2}g/\left(\sigma^{2}g+\left(\sigma^{2}e/r\right)\right)$$

Where σ^²g^ represents genotypic variance, σ²_e_ represents error variance, and r denotes the number of replications.

Multivariate trait relationships were examined through principal component analysis (PCA) using the FactoMineR, factoextra, and ggplot2 packages in R, with all variables standardised to z-scores prior to analysis. Cluster analysis was performed using the K-means clustering algorithm on the standardised dataset with four predefined clusters (k = 4) and 25 random initialisations (nstart = 25). The resulting clusters were visualised on the PCA biplot using the first two principal components. Pearson correlation matrices were generated using the corrplot package. Radar plots were constructed using min–max normalised trait values to visualise multi-trait profiles of contrasting accessions.

## 3 Results

The impact of heat stress on common bean was evident across all five trait categories, as documented in **Figures 2**–**11**, **Tables 2**–**5**; **Supplementary Figures 1–9**, **Supplementary Tables 1–3**. Observable effects on vegetative tissues, such as leaf chlorosis, necrosis, scorching, and tip burning, as well as contrasting growth and yield responses between tolerant and sensitive accessions, are depicted in **Figure 2**. The effects on reproductive structures, including flower and pod abortion, anther integrity, pollen characteristics, stigma receptivity, and ovule condition in contrasting accessions are illustrated in **Figure 3**.

**Figure 2:**
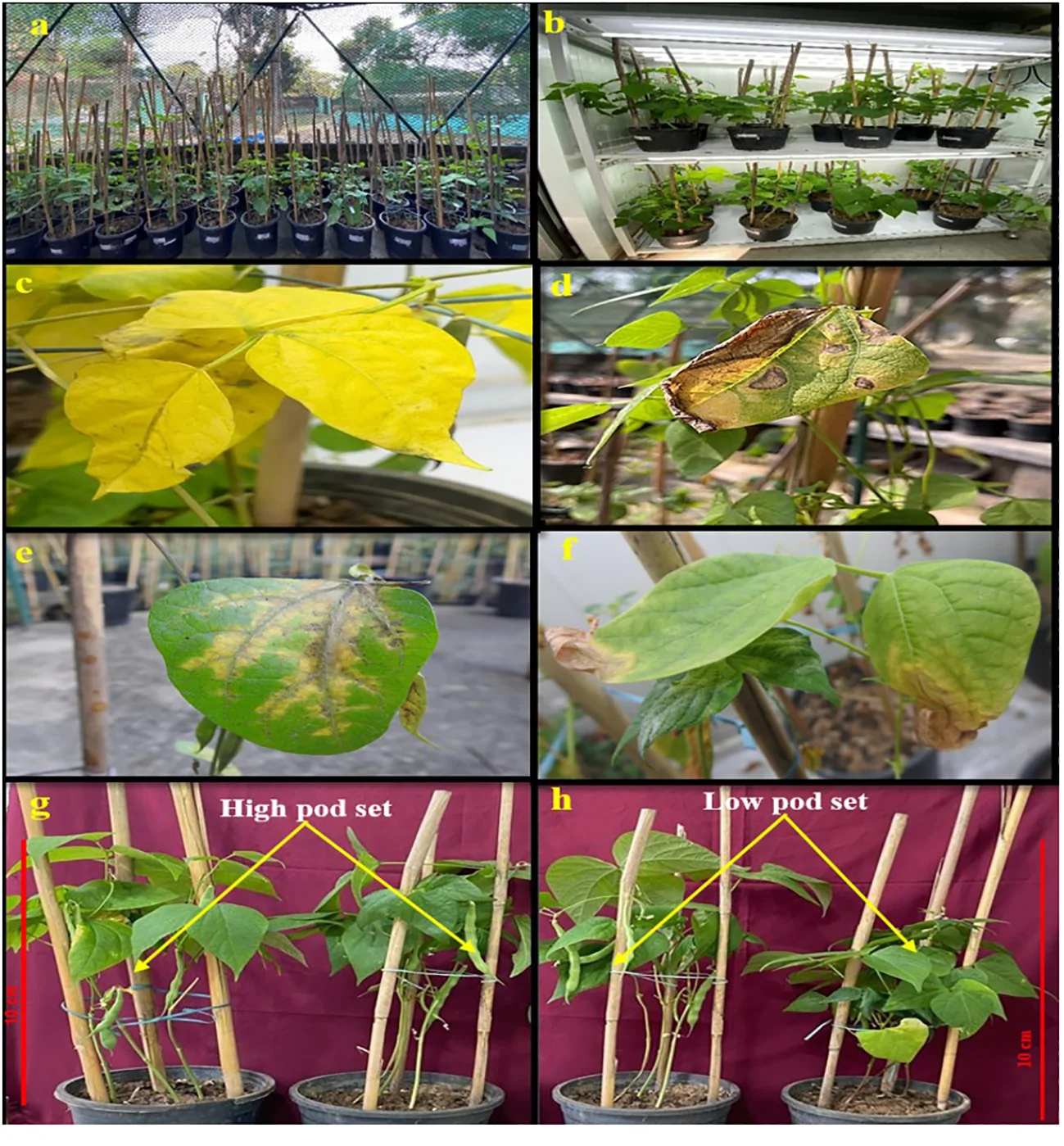
Common bean crop grown under **(a)** outdoor environment and **(b)** growth chamber conditions. Morphological effects of heat stress on common bean accessions: leaf chlorosis **(c)**, leaf necrosis **(d)**, leaf scorching **(e)**, and leaf tip burning **(f)**. Contrasting yield responses under heat stress: high yield in tolerant accessions and poor yield in sensitive accessions **(g, h)**. Scale bar: 10 cm **(g, h)**.

**Figure 3:**
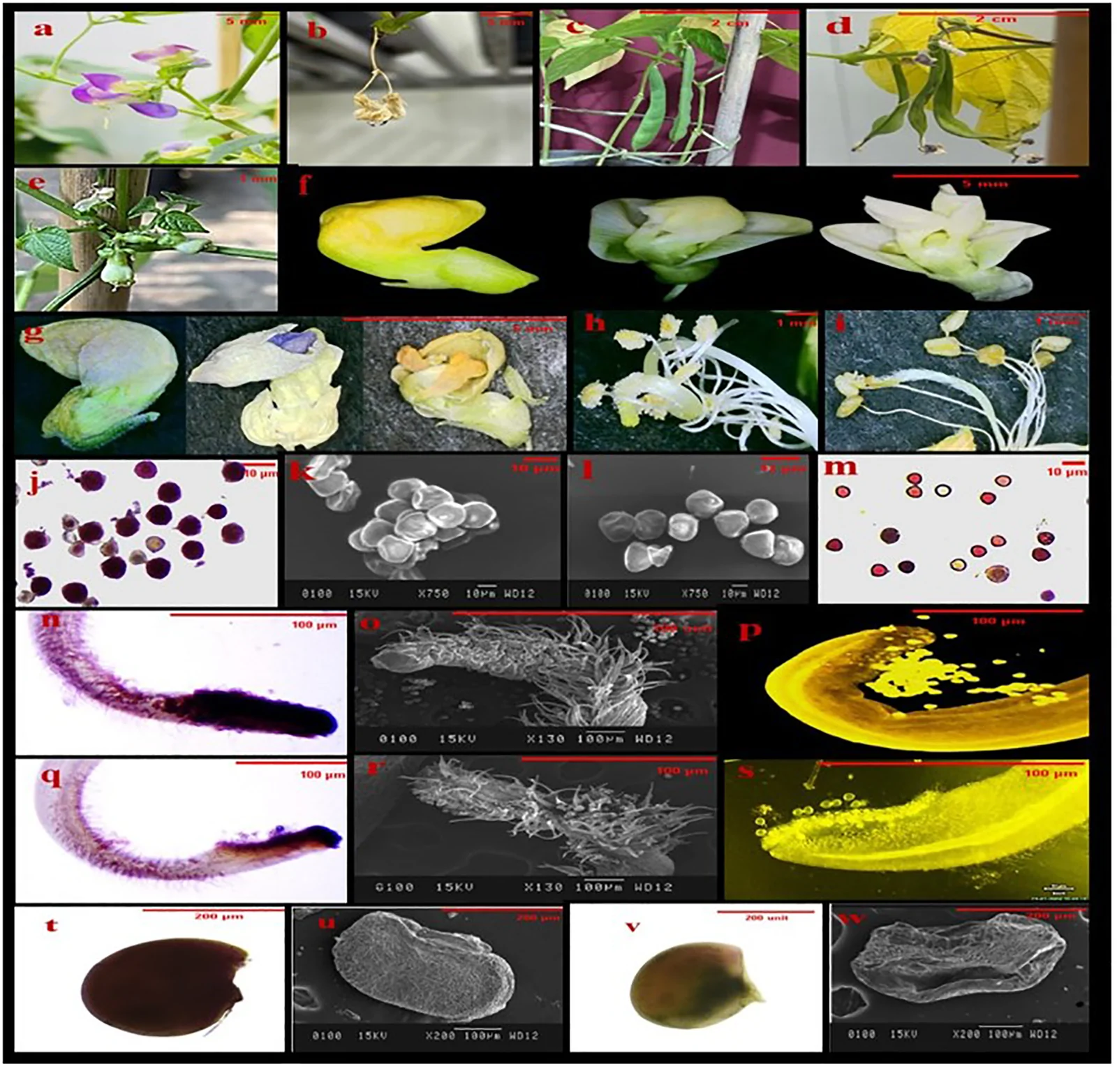
Effects of heat stress on the reproductive phase of common bean. Heat-tolerant accessions under heat stress show: healthy flower **(a)**, healthy pod **(c)**, normal flower developmental stages **(f)**, abundant viable pollen grains **(j, k)**, healthy anther dehiscence **(h)**, highly receptive stigma **(n)**, dense pollen load **(o, p)**, and viable ovules **(t, u)**. Heat-sensitive accessions under heat stress show: aborted flower **(b)**, aborted pods **(d)**, exposed anther **(e)**, stages of aborted flower development **(g)**, distorted anthers **(i)**, non-viable pollen grains **(l, m)**, low stigma receptivity **(q)**, sparse pollen load **(r, s)**, and non-viable distorted ovules **(v, w)**. Scale bars: 5 mm for flowers **(a,b, f, g)**; 1 mm for anthers **(e, h, i)**; 10 µm for pollen grains **(j–m)**; 100 µm for stigma **(n–s)**; 200 µm for ovules **(t–w)**; 2 cm for pods **(c, d)**.

**Figure 4:**
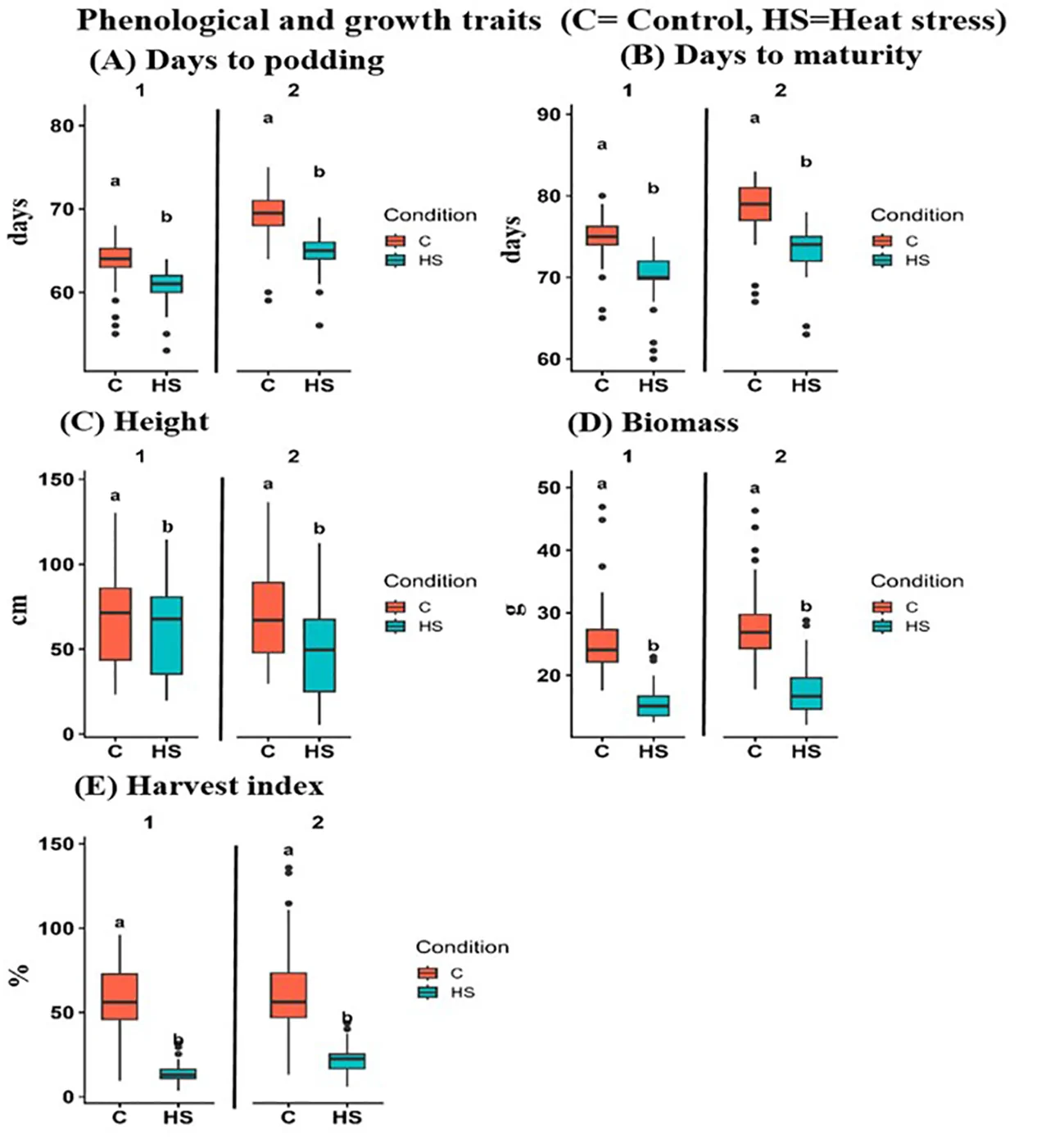
Effects of heat stress on phenological and growth traits in common bean accessions. Box plots showing the distribution of **(A)** days to podding (days), **(B)** days to maturity (days), **(C)** height (cm), **(D)** biomass (g), and **(E)** harvest index (%) under control and heat stress conditions across both growing seasons. Data represent three biological replicates per accession per treatment. Data were analysed using two-way ANOVA to assess the effects of accession and treatment, followed by Tukey’s test for mean separation. Different letters indicate statistically significant differences at p< 0.05.

**Figure 5:**
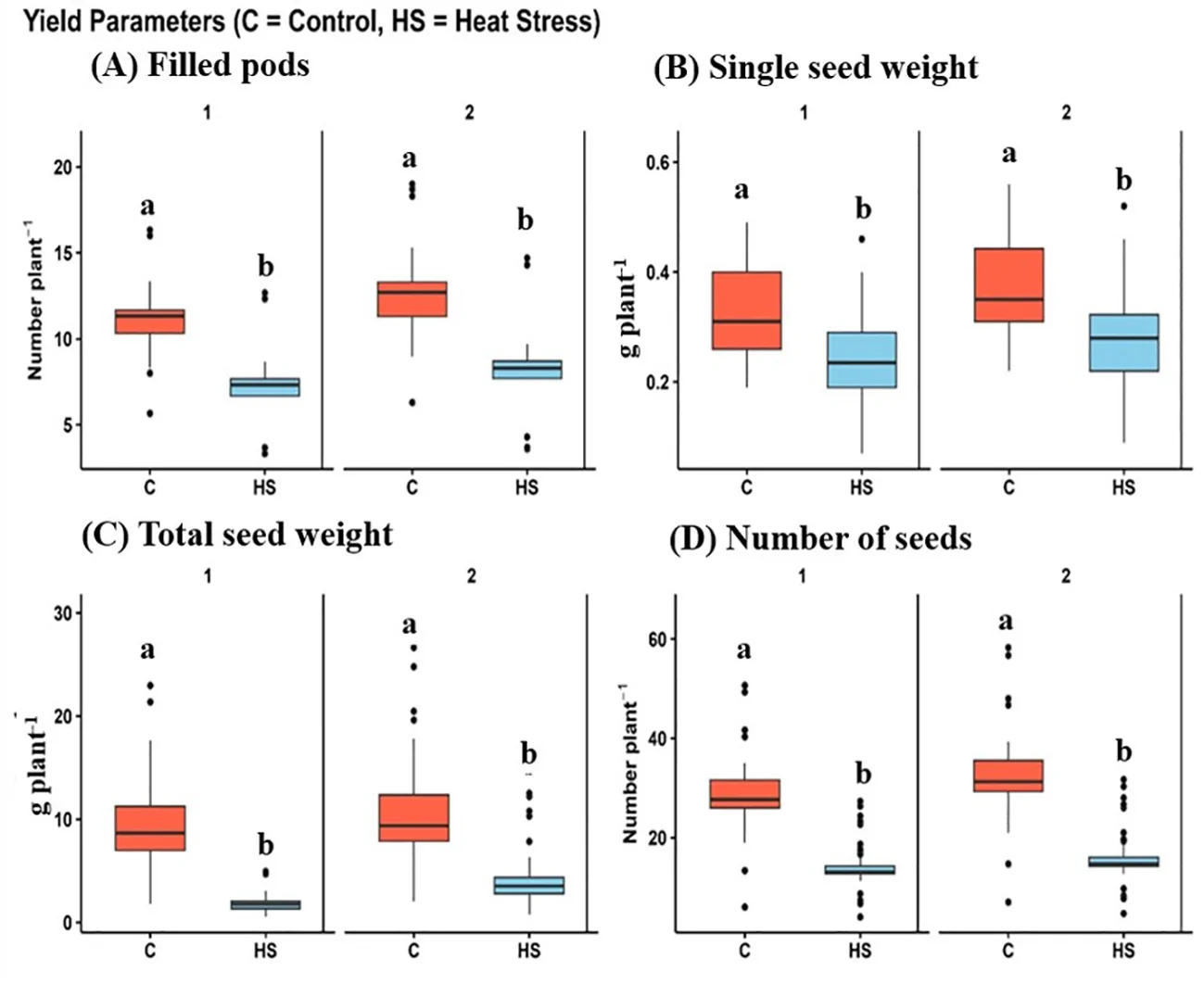
Yield component responses to heat stress in common bean accessions. Box plots showing the distribution of **(A)** filled pods (number plant^-^¹), **(B)** single seed weight (g), **(C)** total seed weight (g plant^-^¹), and **(D)** number of seeds (number plant^-^¹) under control and heat stress conditions across both growing seasons. Data represent three biological replicates per accession per treatment. Data were analysed using two-way ANOVA to assess the effects of accession and treatment, followed by Tukey’s test for mean separation. Different letters indicate statistically significant differences at p< 0.05.

**Figure 6:**
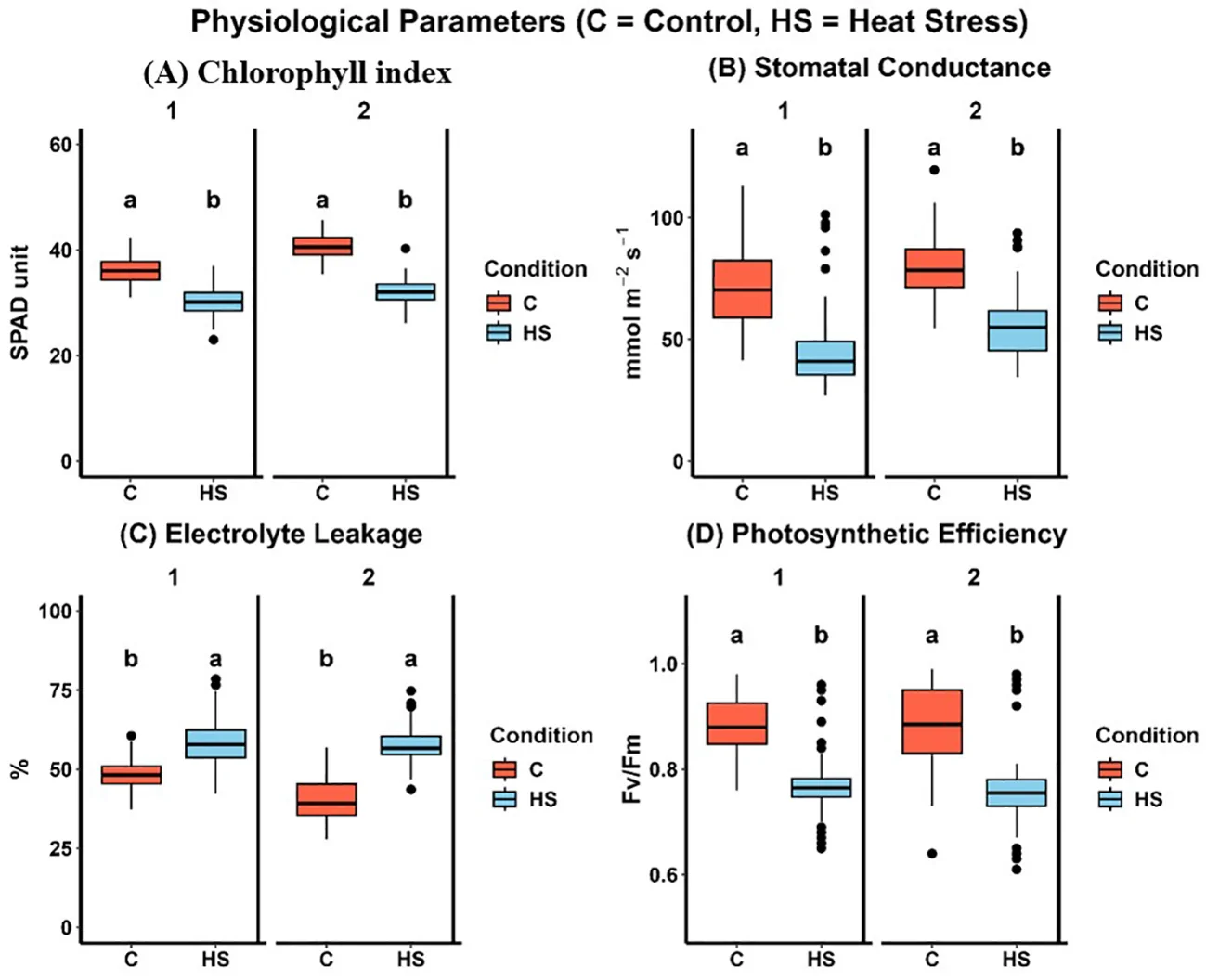
Effects of heat stress on physiological traits in common bean accessions. Box plots showing the distribution of **(A)** chlorophyll index (SPAD units), **(B)** stomatal conductance (mmol m^-^² s^-^¹), **(C)** electrolyte leakage (%), and **(D)** photosynthetic efficiency (Fv/Fm ratio) under control and heat stress conditions across both growing seasons. Data represent three biological replicates per accession per treatment. Data were analysed using two-way ANOVA to assess the effects of accession and treatment, followed by Tukey’s test for mean separation. Different letters indicate statistically significant differences at p< 0.05.

**Figure 7:**
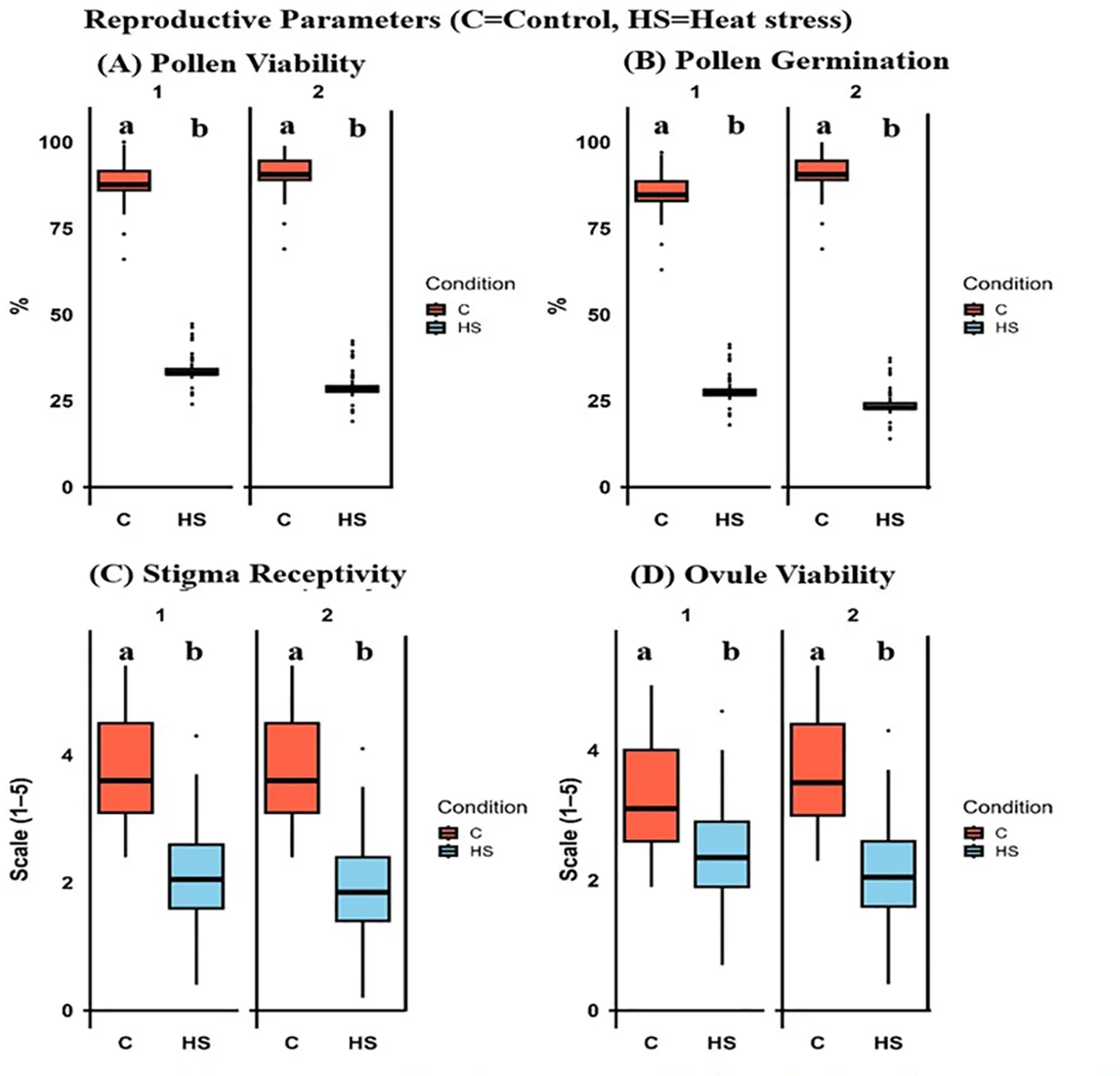
Heat stress effects on reproductive traits in common bean accessions. Box plots illustrating the response of **(A)** pollen viability (%), **(B)** pollen germination (%), **(C)** stigma receptivity (scored on a 1–5 scale based on esterase activity), and **(D)** ovule viability (scored on a 1–5 scale) under control and heat stress conditions across both growing seasons. Data represent three biological replicates per accession per treatment. Data were analysed using two-way ANOVA to assess the effects of accession and treatment, followed by Tukey’s test for mean separation. Different letters indicate statistically significant differences at p< 0.05.

**Figure 8:**
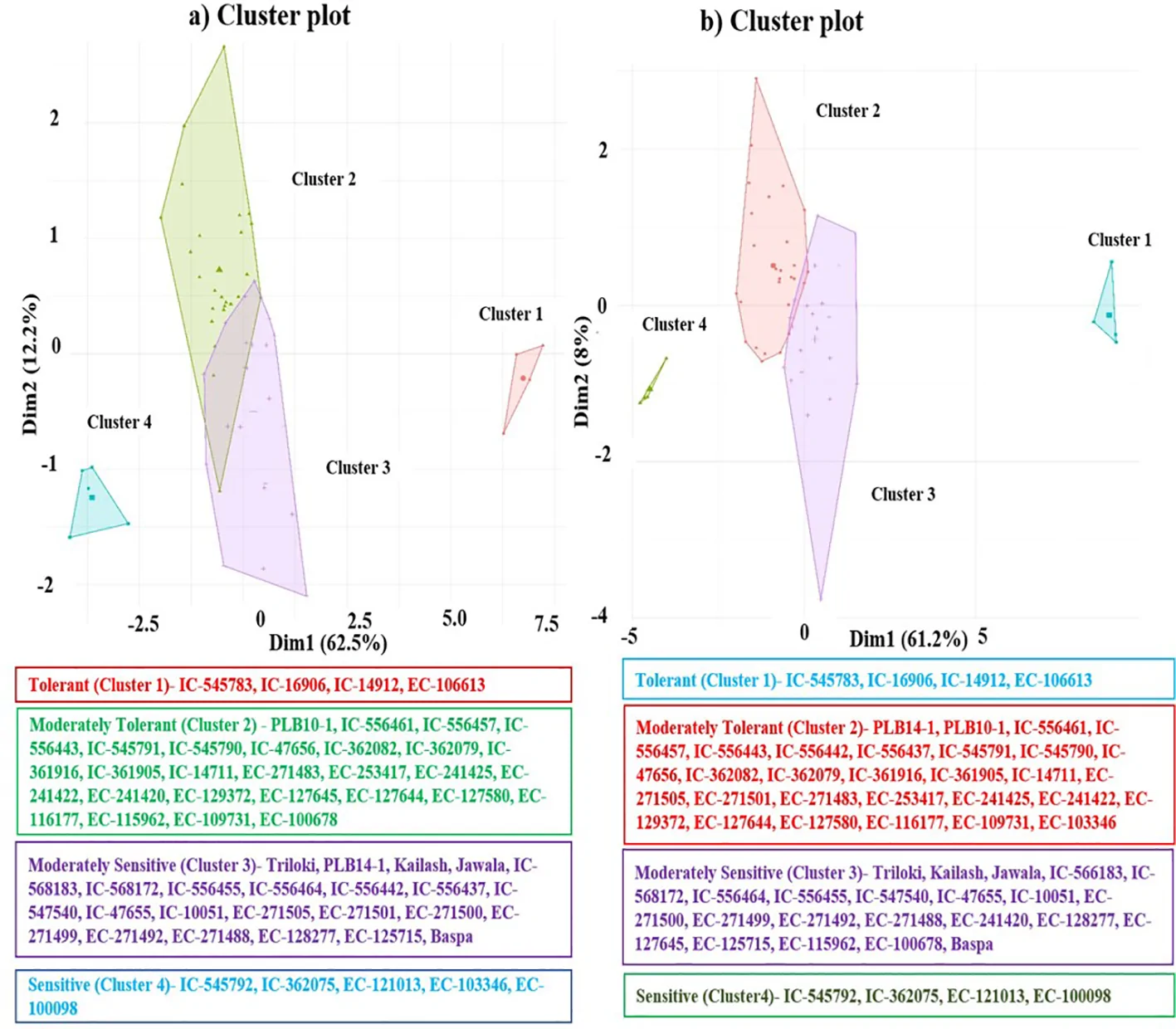
Cluster analysis based on seed yield classifying 56 common bean accessions into contrasting heat sensitivity groups for the **(a)** 2023 and **(b)** 2024 growing seasons. Accessions were assigned to four clusters: Cluster 1 (heat-tolerant), Cluster 2 (moderately tolerant), Cluster 3 (moderately sensitive), and Cluster 4 (heat-sensitive) under heat stress conditions.

**Figure 9:**
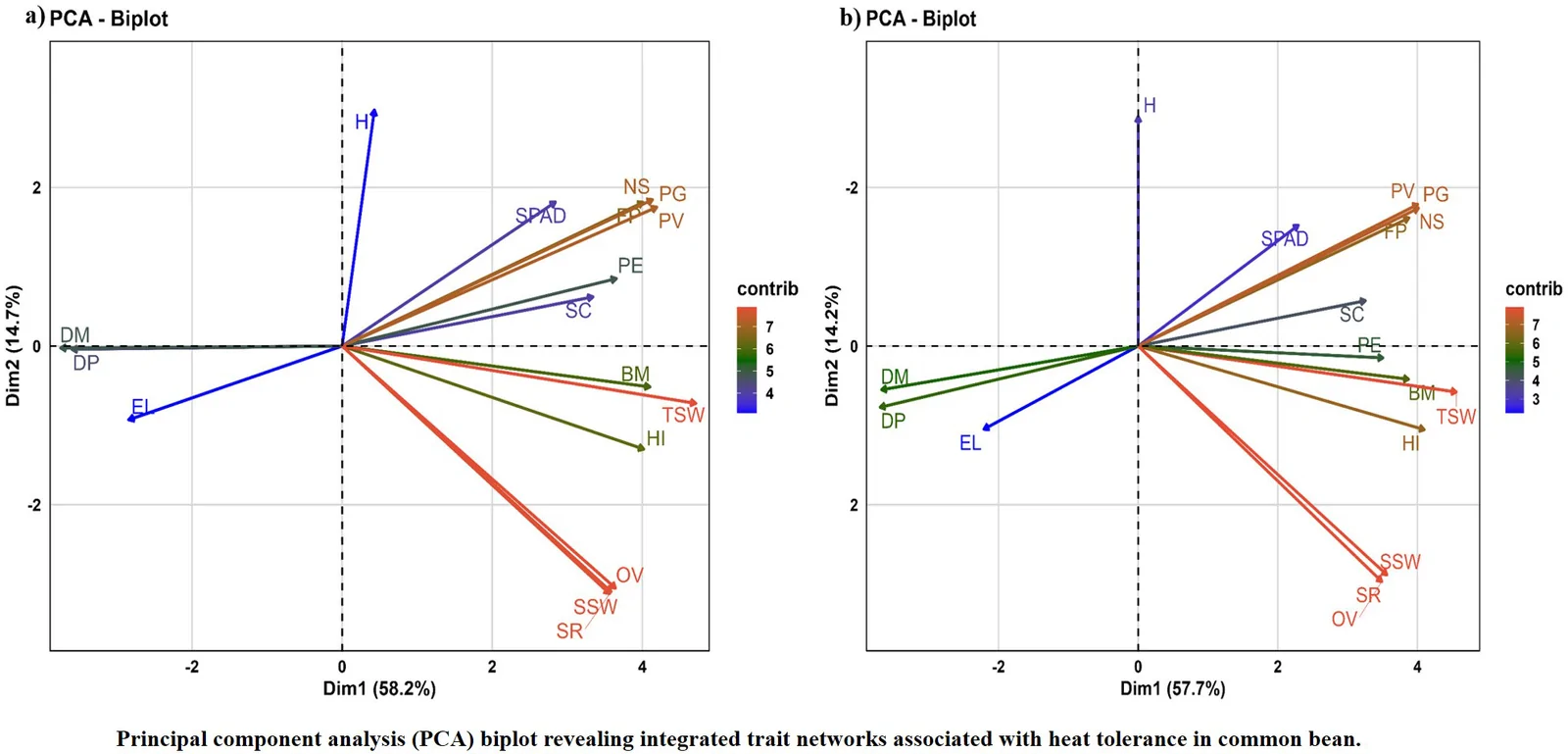
Principal component analysis (PCA) biplot revealing integrated trait networks associated with heat tolerance in common bean. PCA biplot for 17 traits in common bean accessions during first year screening [2023; **(a)**] and second year screening [2024; **(b)**]. Traits included: phenological: days to podding (DP; days) and days to maturity (DM; days); growth: harvest index (HI; %), plant height (H; cm), and biomass (BM; g plant^-^¹); yield: filled pods (FP; number plant^-^¹), number of seeds (NS; number plant^-^¹), single seed weight (SSW; g), and total seed weight (TSW; g plant^-^¹); physiological: electrolyte leakage (EL; %), stomatal conductance (SC; mmol m^-^² s^-^¹), SPAD chlorophyll index (SPAD units), and photosynthetic efficiency (PE; Fv/Fm ratio); and reproductive: pollen viability (PV; %), pollen germination (PG; %), stigma receptivity (SR; scale 1–5), and ovule viability (OV; scale 1–5).

**Figure 10:**
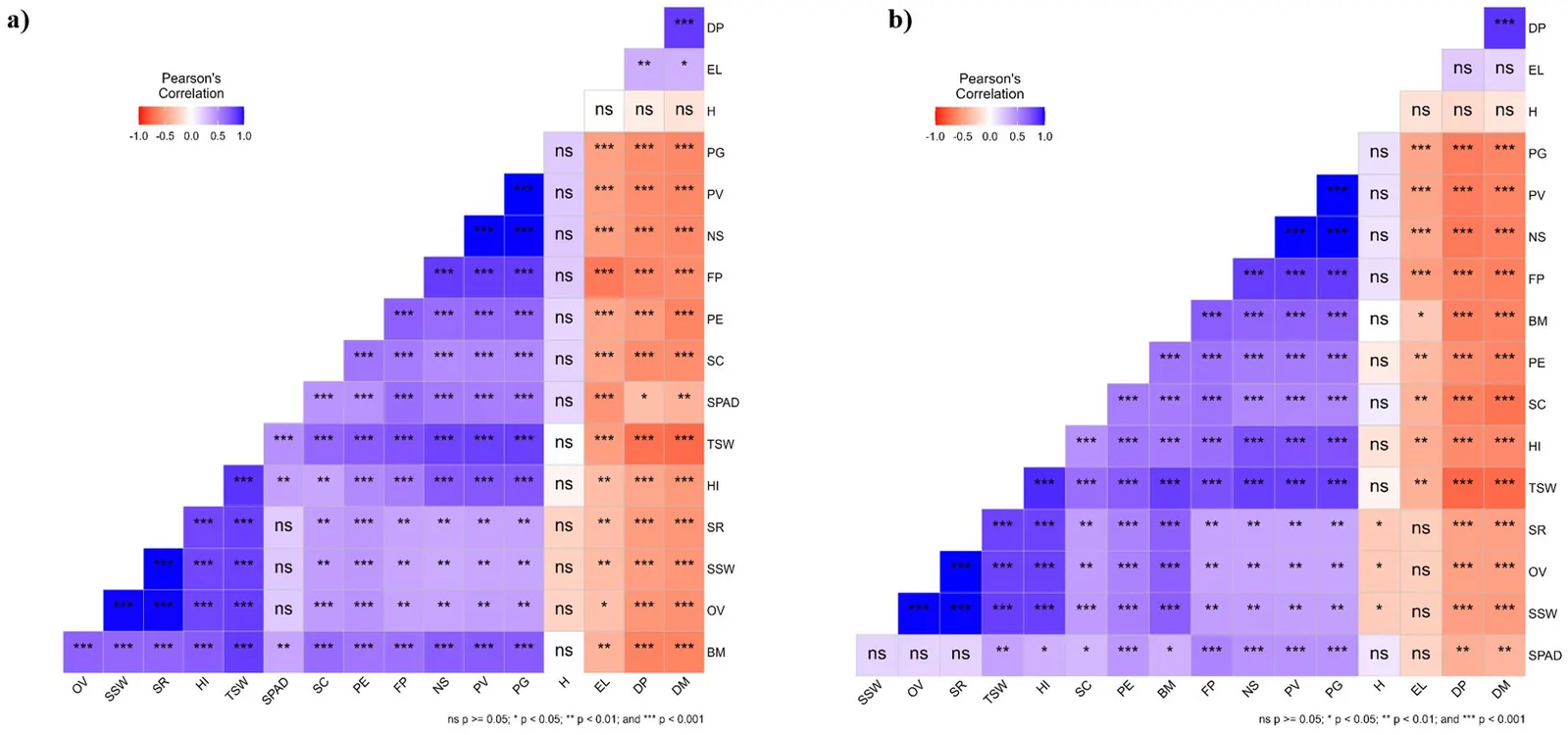
Pearson correlation matrix showing interrelationships amongst physiological, reproductive, and yield traits under heat stress in common bean accessions. Pearson correlation matrix for 17 traits in common bean accessions during first year screening [2023; **(a)**] and second year screening [2024; **(b)**]. Colour gradient represents the strength and direction of correlation, where red indicates negative correlations, blue indicates positive correlations, and lighter shades represent weaker correlations (r values closer to zero). Correlation coefficients range from −1 (perfect negative correlation) to +1 (perfect positive correlation). Significance levels are indicated by asterisks: ns (p ≥ 0.05), * (p< 0.05), ** (p< 0.01), and *** (p< 0.001). Traits included: phenological: days to podding (DP; days) and days to maturity (DM; days); growth: harvest index (HI; %), plant height (H; cm), and biomass (BM; g plant^-^¹); yield: filled pods (FP; number plant^-^¹), number of seeds (NS; number plant^-^¹), single seed weight (SSW; g), and total seed weight (TSW; g plant^-^¹); physiological: electrolyte leakage (EL; %), stomatal conductance (SC; mmol m^-^² s^-^¹), SPAD chlorophyll index (SPAD units), and photosynthetic efficiency (PE; Fv/Fm ratio); and reproductive: pollen viability (PV; %), pollen germination (PG; %), stigma receptivity (SR; scale 1–5), and ovule viability (OV; scale 1–5).

**Figure 11:**
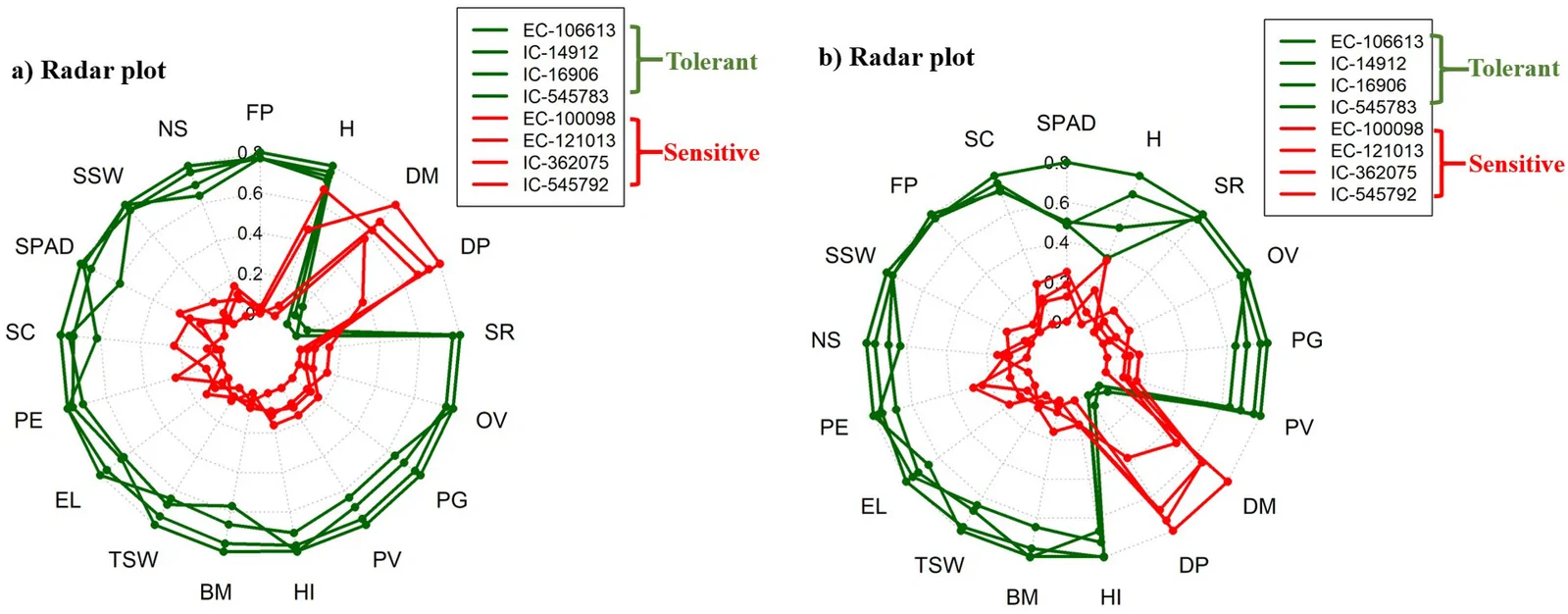
Radar plots for the year 2023 **(a)** and year 2024 **(b)** growing seasons showing the normalised mean values (min–max scaled, 0–1 range) of selected accessions under heat stress. Traits included: phenological: days to podding (DP; days) and days to maturity (DM; days); growth: harvest index (HI; %), plant height (H; cm), and biomass (BM; g plant^-^¹); yield: filled pods (FP; number plant^-^¹), number of seeds (NS; number plant^-^¹), single seed weight (SSW; g), and total seed weight (TSW; g plant^-^¹); physiological: electrolyte leakage (EL; %), stomatal conductance (SC; mmol m^-^² s^-^¹), SPAD chlorophyll index (SPAD units), and photosynthetic efficiency (PE; Fv/Fm ratio); and reproductive: pollen viability (PV; %), pollen germination (PG; %), stigma receptivity (SR; scale 1–5), and ovule viability (OV; scale 1–5). Each radial axis represents one trait, and the distance from the centre indicates the normalised value (scaled between 0 and 1). Different coloured lines correspond to tolerant (green) and sensitive (red) accessions. For phenological traits (DP and DM), higher normalised values indicate delayed development under heat stress.

**Table 2:** Percentage changes in phenological, growth, yield, physiological, and reproductive traits in heat-tolerant and heat-sensitive accessions of common bean (*Phaseolus vulgaris* L.) under control (25/15 °C day/night) and heat stress (32/22 °C day/night) conditions during the reproductive stage (year 2023).

Traits	Heat tolerant accessions		% change	Heat sensitive accessions		% change
	Control	Heat stress		Control	Heat stress	
Phenological traits						
DP	58.9	55.5	-5.7	69.91	65.5	-6.3
DM	65.25	61	-6.5	77	71.75	-6.8
Growth traits						
HI	55.42	42.53	-23.25	21.55	9.8	-54.40
H	84.95	73.625	-13.33	54.54	44.20	-18.9
BM	35.46	23.42	-33.95	20.80	13.42	-35.45
Yield traits						
FP	16.16	12.4	-23.19	9.25	3.5	-62.16
NS	45.5	25.1	-44.69	14.9	6.6	-55.29
SSW	0.43	0.39	-8.13	0.3	0.19	-34.16
TSW	19.73	9.96	-49.50	4.51	1.31	-70.89
Physiological traits						
EL	41.17	45.79	11.22	52.49	75.57	43.96
SC	105.1	95.3	-9.38	60.4	42	-30.46
SPAD	38.25	35.72	-6.61	33.40	25.50	-23.6
PE	0.97	0.95	-2.8	0.805	0.69	-14.2
Reproductive traits						
PV	99.58	45.17	-54.65	74.92	26.67	-64.4
PG	96.58	39.17	-59.45	71.92	20.67	-71.26
SR	5	3.65	-27	3.50	1.68	-52.14
OV	5	3.95	-21	3.00	1.98	-34.17

Traits included: phenological: days to podding (DP; days) and days to maturity (DM; days); growth: harvest index (HI; %), plant height (H; cm), and biomass (BM; g plant^-^¹); yield: filled pods (FP; number plant^-^¹), number of seeds (NS; number plant^-^¹), single seed weight (SSW; g), and total seed weight (TSW; g plant^-^¹); physiological: electrolyte leakage (EL; %), stomatal conductance (SC; mmol m^-^² s^-^¹), SPAD chlorophyll index (SPAD units), and photosynthetic efficiency (PE; Fv/Fm ratio); and reproductive: pollen viability (PV; %), pollen germination (PG; %), stigma receptivity (SR; scale 1–5), and ovule viability (OV; scale 1–5).

Percentage change was calculated as [(Heat stress − Control)/Control] × 100; negative values indicate a decrease under heat stress, whilst positive values indicate an increase.

**Table 3:** Percentage changes in phenological, growth, yield, physiological, and reproductive traits in heat-tolerant and heat-sensitive accessions of common bean (*Phaseolus vulgaris* L.) under control (25/15 °C day/night) and heat stress (32/22 °C day/night) conditions during the reproductive stage (year 2024).

Traits	Heat tolerant accessions		% change	Heat sensitive accessions		% change
	Control	Heat stress		Control	Heat stress	
Phenological traits						
DP	59.5	55.5	-6.722	70.5	65.5	-7.09
DM	68	63.7	-6.32	80.5	75	-6.8
Growth traits						
HI	54.72	41.20	-24.69	23.25	10.40	-55.24
H	79.54	61.95	-22.10	67.29	51.45	-23.52
BM	41.68	27.80	-33.29	21.13	13.87	-34.34
Yield traits						
FP	18.7	14.4	-23	10.4	3.8	-63
NS	52.4	29	-44	16.6	7.6	-54.22
SSW	0.49	0.45	-8.12	0.34	0.23	-34.12
TSW	22.89	11.46	-49.9	5.10	1.45	-71.5
Physiological traits						
EL	44.99	50.73	12.75	48.52	68.9	42.04
SC	100.73	90	-10.64	70.18	45.09	-35.75
SPAD	41.04	36.4	-11.2	37.87	29.31	-22.6
PE	0.98	0.95	-3.51	0.82	0.69	-15.5
Reproductive traits						
PV	99.00	40.17	-59.43	77.92	21.67	-72.19
PG	100	35.17	-64.84	77.92	16.67	-78.61
SR	5	3.45	-31	3.50	1.48	-57.85
OV	5	3.65	-27	3.40	1.68	-50.73

Traits included: phenological: days to podding (DP; days) and days to maturity (DM; days); growth: harvest index (HI; %), plant height (H; cm), and biomass (BM; g plant^-^¹); yield: filled pods (FP; number plant^-^¹), number of seeds (NS; number plant^-^¹), single seed weight (SSW; g), and total seed weight (TSW; g plant^-^¹); physiological: electrolyte leakage (EL; %), stomatal conductance (SC; mmol m^-^² s^-^¹), SPAD chlorophyll index (SPAD units), and photosynthetic efficiency (PE; Fv/Fm ratio); and reproductive: pollen viability (PV; %), pollen germination (PG; %), stigma receptivity (SR; scale 1–5), and ovule viability (OV; scale 1–5).

Percentage change was calculated as [(Heat stress − Control)/Control] × 100; negative values indicate a decrease under heat stress, whilst positive values indicate an increase.

**Table 4:** Mean values and ranges under heat stress (HS), and broad-sense heritability estimates (H²) under heat stress conditions for phenological, growth, yield, physiological, and reproductive traits during two screening years.

Variable	Mean (HS)		Range (HS)		Heritability (H²)	
	1^st^ year	2^nd^ year	1^st^ year	2^nd^ year	1^st^ year	2^nd^ year
Phenological traits						
DP	60	64	69-55	70-56	91.0	92.0
DM	70	73	75-60	78-63	90.0	89.0
Growth traits						
HI	22.20	21.98	47.85-5.43	43.71-6.05	87	89
H	55.44	48.26	120.6-13.66	115.6-7.66	87.70	86.0
BM	15.57	17.58	22.97-12.49	28.82-12.07	95.31	96.2
Yield trait						
FP	7	8	13-3	15-4	85.98	92.37
NS	14	16	27-4	32-5	89.47	88
SSW	0.25	0.29	0.46-0.07	0.52-0.09	91.43	90.71
TSW	3.61	4.06	10.99-0.73	12.53-0.8	93.14	94.48
Physiological traits						
EL	58.83	57.97	78.48-42.19	74.76-43.58	84.85	90.00
SC	46.77	55.29	101.23-27	93.60-34.33	61.22	50.3
SPAD	30.35	32.15	37.00-23	40.27-26.13	48.72	47.2
PE	0.77	0.76	0.96-0.65	0.98-0.61	84.56	85.9
Reproductive traits						
PV	34.08	29.08	47.33-24	42.33-19	69.10	63.8
PG	28.08	24.08	41.33-18	37.33-14	68.43	64.4
SR	2.17	1.97	4.3-0.4	4.1-0.2	60.0	67.6
OV	2.47	2.17	4.6-0.7	4.3-0.4	60.2	72.0

Traits included: phenological: days to podding (DP; days) and days to maturity (DM; days); growth: harvest index (HI; %), plant height (H; cm), and biomass (BM; g plant^-^¹); yield: filled pods (FP; number plant^-^¹), number of seeds (NS; number plant^-^¹), single seed weight (SSW; g), and total seed weight (TSW; g plant^-^¹); physiological: electrolyte leakage (EL; %), stomatal conductance (SC; mmol m^-^² s^-^¹), SPAD chlorophyll index (SPAD units), and photosynthetic efficiency (PE; Fv/Fm ratio); and reproductive: pollen viability (PV; %), pollen germination (PG; %), stigma receptivity (SR; scale 1–5), and ovule viability (OV; scale 1–5).

Heritability categories: Low (< 30), Moderate (30-60), moderately high (60-80), High (> 80).

**Table 5:** Regression equations and coefficients of determination (R²) for the relationships between heat-tolerance residuals (Ys − Ŷs) and different traits during the first and second experimental years.

Traits	Regression equation (First year)	R² (First year)	Regression equation (Second year)	R² (Second year)
Phenological traits				
DF	Residuals = 127.445 − 6.002x + 0.070x²	0.023	Residuals = 124.827 − 5.542x + 0.061 x²	0.049
DP	Residuals = 281.587 − 9.432x + 0.079x²	0.059	Residuals = 101.553 – 2.734x + 0.018x²	0.068
DM	Residuals = 155.15 − 4.192x + 0.028x²	0.078	Residuals = 180.008 − 4.669x + 0.03x²	0.064
Growth traits				
H	Residuals = −6.113 + 6.672x	0.013	Residuals = 3.484 – 5.499x	0.026
BM	Residuals = −14.662 + 23.437x	0.273	Residuals = −11.237 + 17.72x	0.064
HI	Residuals = −12.439 + 51.817x	0.818	Residuals = −15.194 + 40.568x	0.704
Yield traits				
FP	Residuals = −2.833 + 6.032x	0.128	Residuals = −3.282 + 8.224x	0.148
NS	Residuals = −4.477 + 9.033x	0.421	Residuals = −20.276 + 42.339x – 2.683x²	0.351
SSW	Residuals = −3.437 + 4.525x	0.522	Residuals = −17.964 + 23.542x	0.523
TSW	Residuals = −3.61 + 9.575x	0.876	Residuals = −15.517 + 40.789x	0.801
Physiological traits				
SPAD	Residuals = 4.745x − 3.983	0.081	Residuals = 3.375x – 2.678	0.026
SC	Residuals = 1.999x − 1.301	0.071	Residuals = 0.839x – 0.586	0.008
PE	Residuals = − 0.608x + 0.534	0.001	Residuals = −1.841x + 1.607	0.019
EL	Residuals = − 0.488x + 0.593	0.003	Residuals = − 0.758x + 1.117	0.018
Reproductive traits				
PV	Residuals = 26.042x − 10.049	0.449	Residuals = 29.003x − 9.255	0.488
PG	Residuals = 5.971x – 0.771x²	0.428	Residuals = 7.25x + 0.79x²	0.460
OV	Residuals = 4.25x – 3.189	0.438	Residuals = 5.114x – 2.981	0.387
SR	Residuals = 4.697x – 2.669	0.428	Residuals = 4.978x – 2.552	0.366

Traits included: phenological: days to podding (DP; days) and days to maturity (DM; days); growth: harvest index (HI; %), plant height (H; cm), and biomass (BM; g plant^-^¹); yield: filled pods (FP; number plant^-^¹), number of seeds (NS; number plant^-^¹), single seed weight (SSW; g), and total seed weight (TSW; g plant^-^¹); physiological: electrolyte leakage (EL; %), stomatal conductance (SC; mmol m^-^² s^-^¹), SPAD chlorophyll index (SPAD units), and photosynthetic efficiency (PE; Fv/Fm ratio); and reproductive: pollen viability (PV; %), pollen germination (PG; %), stigma receptivity (SR; scale 1–5), and ovule viability (OV; scale 1–5).

### 3.1 Yield-based regression analysis for identifying heat tolerance

Across both screening years, a significant positive linear correlation was identified between seed yield under heat stress (Ys) and seed yield under control conditions (Yc) in the present study. The regression equations were Ŷs = 0.441Yc − 0.573 (R² = 0.663) for the first year and Ŷs = 0.438Yc − 0.581 (R² = 0.655) for the second year (**Supplementary Figures 1a, b**). The heat-tolerance residuals (R = Ys − Ŷs), derived from these equations, exhibited a strong positive correlation with the yield ratio (Ys/Yc; expressed as total seed weight under heat stress relative to control), described by R = 9.575(Ys/Yc) − 3.61 (R² = 0.876) in the first year and R = 10.234(Ys/Yc) − 3.893 (R² = 0.877) in the second year (**Supplementary Figures 1c, d**). These residuals were used as the primary metric for assessing heat tolerance in all subsequent analyses.

### 3.2 Trait responses to heat stress

#### 3.2.1 Phenological traits

Phenological traits exhibited substantial variation amongst the accessions under both experimental conditions. Analysis of variance (ANOVA) revealed significant effects of accession, treatment, and their interactions for most comparisons (**Supplementary Table 1**). Box plot analysis indicated a shift towards lower median values under heat stress in both screening years, signifying an advancement in podding and maturity across all accessions (**Figure 4**). Heat-sensitive accessions, including IC-545792, EC-100098, EC-121013, and IC-362075, demonstrated substantially longer absolute durations for both days to podding and days to maturity under control conditions than heat-tolerant accessions IC-14912, EC-106613, IC-545783, and IC-16906. However, the proportional advancement under heat stress was broadly comparable across both groups in both the years. Comprehensive trait values and percentage changes for both groups across both years are presented in **Tables 2**, **3**, respectively.

#### 3.2.2 Growth traits

Analysis of variance (ANOVA) indicated significant effects of accession and treatment on all three growth traits, with the interaction between accession and treatment being significant for certain traits (**Supplementary Table 1**). Box plot analysis corroborated the reductions in the median values for plant height, biomass, and harvest index under heat stress across both years (**Figure 4**). Heat-tolerant accessions (IC-14912, EC-106613, IC-545783, and IC-16906) exhibited comparatively smaller reductions than heat-sensitive accessions (IC-545792, EC-100098, EC-121013, and IC-362075) across all three traits, with the most pronounced group contrast observed for the harvest index, which declined by 23.25% and 24.69% in tolerant accessions compared to 54.40% and 55.24% in sensitive accessions in the first and second years, respectively. Plant height showed broadly comparable reductions between the groups in both years. Biomass declined to a similar extent in both groups in both years. Complete trait values and percentage changes are provided in **Tables 2**, **3**, respectively, and broad-sense heritability estimates under heat stress are presented in **Table 4**.

#### 3.2.3 Yield traits

Yield attributes exhibited significant variation amongst accessions under both treatments, with the accession × treatment interaction being significant for number of seeds (NS), single-seed weight (SSW), and total seed weight (TSW) (**Supplementary Table 1**). Box plots demonstrated a shift towards lower median values and increased variability under heat stress for all four yield traits in both years (**Figure 5**). Heat-tolerant accessions (IC-14912, EC-106613, IC-545783, and IC-16906) experienced substantially smaller reductions in all yield components than heat-sensitive accessions (IC-545792, EC-100098, EC-121013, and IC-362075). Single seed weight showed the smallest absolute reduction amongst all yield components in tolerant accessions, declining by only ~8% compared with ~34% in sensitive accessions, demonstrating a superior capacity for seed filling retention under stress, and its strong regression association with heat tolerance residuals R^2^ = 0.522 and 0.523 confirms it as a reliable agronomic indicator of genotypic heat tolerance (Kumar et al., 2006). The number of filled pods exhibited larger absolute reductions in sensitive accessions across both years. Full trait values and percentage changes for all yield components are presented in **Tables 2**, **3**, respectively. Broad-sense heritability estimates for yield traits under heat stress exceeded 85% for all four components (**Table 4**).

#### 3.2.4 Physiological traits

Analysis of variance (ANOVA) demonstrated significant effects of accession and treatment on most physiological traits, with accession × treatment interactions exhibiting variability across traits and years (**Supplementary Table 1**). Box plot analysis corroborated the distinct differences in the distribution of physiological trait values between tolerant and sensitive accessions under heat stress conditions (**Figure 6**).

Electrolyte leakage increased under heat stress across all accessions, with tolerant accessions exhibiting increases of 11.2% and 12.7%, in contrast to the substantially larger increases of 43.9% and 42.4% observed in sensitive accessions over the two-year period.

Stomatal conductance, SPAD chlorophyll values, and photosynthetic efficiency all declined under heat stress, with heat-tolerant accessions consistently showing smaller reductions than sensitive ones across both years for all three parameters.

Comprehensive trait values and percentage changes are presented in **Tables 2**, **3**. The heritability estimates for electrolyte leakage and photosynthetic efficiency exceeded 84%, whereas stomatal conductance and SPAD values exhibited moderate heritability across both years (**Table 4**).

#### 3.2.5 Reproductive traits

All four reproductive traits exhibited significant variation amongst accessions and demonstrated notable accession × treatment interactions in both years (**Supplementary Table 1**). Box plots revealed a downward shift in median values and increased variability under heat stress compared to that under control conditions (**Figure 7**).

In heat-tolerant accessions (IC-14912, EC-106613, IC-545783, and IC-16906), pollen viability decreased by 54.65% and 59.43%, pollen germination by 59.45% and 64.84%, stigma receptivity by 27.0% and 31.0%, and ovule viability by 21.0% and 27.0% in the first and second years, respectively. Conversely, in heat-sensitive accessions (IC-545792, EC-100098, EC-121013, and IC-362075), pollen viability declined by 64.4% and 72.19%, pollen germination by 71.26% and 78.61%, stigma receptivity by 52.14% and 57.85%, and ovule viability by 34.17% and 50.73%, respectively, over the two years (**Tables 2**, **3**). The broad-sense heritability estimates for reproductive traits under heat stress ranged from 60% to 72% across both years (**Table 4**).

#### 3.2.6 Regression analysis of trait associations with heat-tolerance residuals

To assess the relative contribution of each trait category to heat tolerance, regression analyses were conducted between heat tolerance residuals (Ys − Ŷs) and trait ratios (heat stress relative to control) across all five categories. The regression equations and corresponding coefficients of determination (R²) for both experimental years are presented in **Table 5**, whereas the regression plots are shown in **Supplementary Figures 2–9**. Phenological traits consistently exhibited low R² values across both years, including days to flowering, days to podding, and days to maturity (**Supplementary Figures 2, 3**).

Growth traits demonstrated variable associations, with plant height ratio and biomass ratio exhibiting low to moderate relationships, whereas the harvest index showed a stronger association with heat tolerance residuals across both years (**Supplementary Figures 2, 3**).

Yield traits exhibited the strongest associations amongst all trait categories, with single seed weight ratio, total seed weight ratio, and harvest index demonstrating strong relationships, whereas seed number ratio showed moderate associations and filled pod ratio exhibited low to moderate relationships across both years (**Supplementary Figures 4, 5**).

Physiological traits consistently exhibited low R² values in both years, including SPAD ratio, stomatal conductance ratio, photosynthetic efficiency ratio, and electrolyte leakage ratio (**Supplementary Figures 6, 7**).

Reproductive traits exhibited the most consistent moderate associations across both years, including pollen viability ratio, pollen germination ratio, ovule viability ratio, and stigma receptivity ratio (**Supplementary Figures 8, 9**).

### 3.3 Cluster analysis for identification of common bean accessions contrasting in heat sensitivity based on yield traits

Cluster analysis of the yield performance under heat stress conditions categorised the 56 accessions into four distinct clusters across both screening years (**Figures 8a, b**). Cluster 1 included accessions that consistently exhibited the highest pod and seed yields under heat stress in both years, specifically IC-14912, EC-106613, IC-545783, and IC-16906. Cluster 2 comprised accessions with moderate tolerance, whilst Cluster 3 consisted of those with moderate sensitivity to heat stress. Cluster 4 encompassed accessions that demonstrated the most pronounced yield reductions under heat stress, namely, IC-545792, EC-100098, EC-121013, and IC-362075, in both years. The configuration of the four clusters and the assignment of accessions within the extreme clusters were consistent across both screening seasons.

### 3.4 Principal component analysis

PCA biplots consistently demonstrated patterns of trait associations under heat stress in both years (**Figures 9a, b**). In the first year, Principal Component 1 (PC1) accounted for 58.2% of the total variance, whereas Principal Component 2 (PC2) explained 14.7%, resulting in a cumulative contribution of 72.9% (**Figure 9a**). In the second year, PC1 accounted for 57.7% and PC2 for 14.2%, with a cumulative contribution of 71.9% (**Figure 9b**). The eigenvalues, percentage variance explained, cumulative variance, and positive and negative variable loadings of all principal components for both years are provided in **Supplementary Tables 2**, **3**. In both years, traits such as SSW, TSW, HI, BM, FP, NS, PV, PG, OV, and SR exhibited strong positive loadings along PC1. In addition, SC, PE, and SPAD were positioned on the positive side of PC1, albeit with comparatively shorter vectors. Conversely, EL, DP, and DM exhibited negative loadings along PC1 in both years. Plant height (H) was primarily aligned along PC2, showing a strong positive loading in both years. Along PC2, OV, SR, and SSW were positioned in the lower quadrant, whereas SPAD and H were located in the upper quadrant in both the years.

### 3.5 Pearson correlation analysis

Pearson’s correlation analysis, conducted separately for both years, demonstrated broadly consistent patterns of trait associations amongst the 56 accessions under heat stress (**Figures 10a, b**). The traits SSW, TSW, BM, HI, FP, and NS were positively and significantly correlated with PV, PG, OV, and SR in both years (p< 0.001). SC, PE, and SPAD were positively and significantly correlated with both yield and reproductive traits, with particularly strong associations observed between SC and PE with FP, NS, PV, and PG. EL displayed significant negative correlations with most yield, reproductive, and physiological traits across both years. DP and DM showed strong and significant negative correlations with yield and reproductive and physiological traits (predominantly p< 0.001). Plant height exhibited predominantly non-significant or weak correlations with yield, reproductive, and physiological traits in both years of the study. The correlation structure remained stable across both screening years, with only minor variations in the magnitude of individual coefficients.

### 3.6 Radar plots

Radar plots for both years effectively differentiated between heat-tolerant and heat-sensitive accessions across the complete set of traits (**Figures 11a, b**). In both years, heat-tolerant accessions consistently demonstrated higher normalised values for HI, BM, SPAD, SC, PV, PG, OV, SR, NS, SSW, and TSW than heat-sensitive accessions. Heat-sensitive accessions exhibited significantly lower values for reproductive and yield-associated traits, with the most notable reductions observed in pollen viability, pollen germination, ovule viability, seed number, and seed weight. Additionally, heat-sensitive accessions displayed higher normalised DP and DM values under heat stress conditions. The radar plot profiles were consistent across both experimental years.

## 4 Discussion

Heat stress experienced during the reproductive phase resulted in significant and quantifiable damage across all five trait categories assessed in this study. The extent of this damage, involving accelerated phenology, suppressed vegetative growth, impaired physiological function, disrupted reproductive development, and reduced seed yield, underscores the particular susceptibility of common bean to elevated temperatures at this growth stage. However, the extent of these effects showed notable and consistent differences amongst accessions over two separate growing seasons, indicating that the variation in heat tolerance due to genotype is both real and stable. The subsequent sections examine each trait category in sequence, interpret the observed patterns, and relate them to the central finding that reproductive function and seed weight stability are the primary determinants of heat tolerance in this germplasm.

### 4.1 Regression-based screening captures stable genotypic variation in heat tolerance

The nearly identical regression parameters observed across both growing seasons, slopes of 0.441 and 0.438, intercepts of −0.573 and −0.581, demonstrate that the relationship between yield potential under control conditions and yield under heat stress was consistent and stable. This consistency reflects stable genotypic variation rather than seasonal artifacts. The residual-based tolerance index (Porch, 2006) successfully disentangled heat tolerance from intrinsic yield potential, thereby avoiding the common misclassification of high-yielding accessions as tolerant merely due to their absolute yield advantage under stress conditions. The high R² values for the residual versus seed yield ratio relationship (0.87 and 0.877) across both years further substantiate the framework as a reliable and reproducible method for ranking common bean germplasm for heat tolerance under controlled conditions.

### 4.2 Phenological acceleration and vegetative growth suppression are general responses shared across accessions

Heat stress accelerated the initiation of podding and maturity across all 56 accessions, a phenomenon frequently observed in grain legumes and generally attributed to the thermogenic stimulation of developmental rates under elevated temperatures (Farooq et al., 2017; Tene et al., 2025; Priya et al., 2025). Although earlier maturity is often considered a stress-avoidance mechanism, the regression data from the present study do not support phenological parameters as significant predictors of heat tolerance, as evidenced by consistently low R² values for days to flowering, podding, and maturity across both years (Section 3.2.6). This indicates that developmental acceleration was a common response amongst both tolerant and sensitive accessions and did not confer a measurable advantage in terms of yield maintenance under stress. Notably, heat-sensitive accessions exhibited substantially longer absolute durations for both phenological traits under control conditions; however, the proportional advancement under heat stress was comparable across groups, suggesting that baseline phenological duration, rather than stress-induced acceleration, differentiates the two groups (Agtunong et al., 1992; Araújo et al., 2015).

Vegetative growth characteristics, including plant height, biomass, and harvest index, were uniformly suppressed under heat stress across all accessions, aligning with the documented inhibitory effects of elevated temperatures on cell division, cell elongation, and assimilate production in common bean and related legumes (De Ron et al., 2016; Vargas et al., 2021; Tene et al., 2025). The reduction in harvest index was significantly more pronounced in heat-sensitive accessions, with declines of 54.40% and 55.24% compared to 23.25% and 24.69% in tolerant accessions over the two-year period, indicating impaired assimilate partitioning towards reproductive organs under stress conditions (Vargas et al., 2021). In contrast, plant height exhibited similar reductions across both groups in both years, and its regression association with heat-tolerance residuals was negligible (R²< 0.030). This finding confirms that plant height serves as a general indicator of overall stress severity rather than a distinguishing feature of heat tolerance. Conversely, the harvest index, which reflects the efficiency of assimilate allocation to reproductive sinks, proved to be more functionally informative and demonstrated the strongest regression association amongst growth traits (R² = 0.818 and 0.704).

### 4.3 Physiological stability supports reproductive function under heat stress

Tolerant accessions consistently demonstrated superior physiological performance under heat stress over both years, as evidenced by reduced electrolyte leakage, increased stomatal conductance, enhanced chlorophyll retention, and more stable photosynthetic efficiency. This suggests that they maintained greater cellular and photosynthetic integrity under elevated temperatures. The observed reduction in chlorophyll content across all accessions corresponds with heat-induced destabilisation of chloroplast membranes, accelerated chlorophyll degradation, and inhibition of key photosynthetic enzymes, leading to diminished photosynthetic efficiency (Chaisompongpan et al., 1990; Srinivasan et al., 1996; Porch and Jahn, 2001; Vargas et al., 2021). The concurrent decline in stomatal conductance under heat stress limited CO_2_ uptake and reduced carbon assimilation, with sensitive accessions experiencing declines of 30.46% and 35.75%, compared to only 9.38% and 10.64% in tolerant lines. Electrolyte leakage, the most direct indicator of membrane damage, increased by 43.96% and 42.04% in sensitive accessions, more than three times the increase observed in tolerant accessions, reflecting substantially greater cellular disruption under thermal stress. Despite these clear group differences in physiological performance, regression analysis against heat-tolerance residuals revealed consistently low R² values for all four physiological parameters across both years (Section 3.2.6). This does not diminish the importance of physiological stability; rather, it indicates that physiological resilience functions as a prerequisite for reproductive competence rather than as a direct predictor of yield outcome in its own right. The strong positive co-variation between physiological parameters and reproductive and yield traits observed in PCA and Pearson’s correlation analyses with SC and PE showing significant positive associations with FP, NS, PV, and PG supports the interpretation that physiological stability underpins reproductive function by sustaining carbon supply and membrane integrity in developing floral and seed tissues. Accessions with better membrane stability and photosynthetic function under heat stress were better positioned to sustain pollen viability, ovule development, and seed set, as reflected in the consistently superior reproductive performance of the four identified tolerant accessions (Soltani et al., 2019). These findings are in agreement with Vargas et al. (2021) and Tene et al. (2025), who similarly noted that physiological parameters differentiate tolerant and sensitive common bean accessions without serving as primary predictors of yield under heat stress.

### 4.4 Reproductive traits are the primary determinants of heat tolerance

The reproductive system was unequivocally identified as the most temperature-sensitive component of common bean development in this study. The ability to maintain reproductive function under heat stress emerged as the most reliable differentiator between tolerant and sensitive accessions. All four reproductive traits: pollen viability, pollen germination, stigma receptivity, and ovule viability experienced substantial declines under heat stress, with the magnitude of decline being notably greater in sensitive accessions across both years. Specifically, pollen viability and pollen germination decreased by up to 72.19% and 78.61%, respectively, in sensitive accessions, compared to 59.43% and 64.84% in tolerant lines. Stigma receptivity and ovule viability exhibited group differences exceeding 20 percentage points in both years. This simultaneous compromise of male and female gametophyte function directly accounts for the severe yield losses in sensitive accessions, as successful fertilisation necessitates the concerted operation of functional pollen, receptive stigma, and viable ovules. High-temperature stress is well established as a major disruptor of pollen viability and tube growth in common bean (Porch and Jahn, 2001; Vargas et al., 2021; Tene et al., 2025) and related beans (Hu et al., 2022; Suh et al., 2023), with cascading consequences for pod set and seed number. These findings align with those of Rose et al. (2023), who reported a decline in pollen viability to 24.9% in the heat-sensitive cultivar Calima under 31/24 °C conditions, whilst a tolerant breeding line retained 71.1% a differential of comparable magnitude to that observed between the contrasting accession groups in the present study. The documented impairment of stigma receptivity and ovule viability further confirms that reproductive vulnerability under heat stress extends beyond pollen to the female reproductive system, consistent with observations in other legumes (Vargas et al., 2021; Tene et al., 2025).

Regression analysis identified reproductive trait ratios as the most consistent predictors of heat-tolerance residuals across both years, with R² values of 0.449 and 0.488 for pollen viability, 0.428 and 0.460 for pollen germination, 0.438 and 0.387 for ovule viability, and 0.428 and 0.366 for stigma receptivity (Section 3.2.6). Coupled with the moderate-to-high broad-sense heritability estimates for these traits (60–72%; **Table 4**), these findings suggest that variation in reproductive resilience under heat stress is predominantly under genetic control and thus amenable to selection in a breeding context (Tene et al., 2023).

The combination of functional relevance, consistent predictive association with yield residuals, and heritable genetic variation renders reproductive traits particularly pollen viability and pollen germination ratios reliable supplementary selection criteria for enhancing heat tolerance in common bean. The strong associations between single seed weight (SSW), total seed weight (TSW), and reproductive trait performance observed in Pearson’s correlation analysis (p< 0.001 in both years) further support this conclusion- seed filling capacity and reproductive integrity are closely coordinated processes under heat stress, and accessions that maintained reproductive function also retained seed weight most effectively. Single seed weight declined by only 8.13% and 8.12% in tolerant accessions compared with 34.16% and 34.12% in sensitive ones, the most pronounced pairwise contrast across all yield components measured and its strong regression association with heat-tolerance residuals (R² = 0.522 and 0.523) confirms it as a reliable agronomic indicator of genotypic heat tolerance (Kumar et al., 2006).

### 4.5 Multi-trait convergence identifies four accessions as priority genetic resources

Cluster analysis, regression residuals, principal component analysis (PCA), and radar plots consistently identified the same four accessions IC-14912, EC-106613, IC-545783, and IC-16906 as superior performers across both growing seasons. These accessions demonstrated a multi-trait profile under heat stress, characterised by minimal declines in all four reproductive traits, the lowest increases in electrolyte leakage, optimal maintenance of chlorophyll content and stomatal conductance, and significantly higher retention of single seed weight and total seed weight. The consistent cluster assignment of these accessions over two years strongly suggests that their thermal resilience is attributable to stable genetic characteristics rather than season-specific performance. In contrast, accessions IC-545792, EC-100098, EC-121013, and IC-362075 were consistently classified within the heat-sensitive cluster across both years, serving as well-characterised sensitive reference lines for future screening studies. All four tolerant accessions originate from the NBPGR gene bank collection, ensuring they are formally conserved, documented, and readily accessible for use in breeding programmes without additional biosafety or regulatory constraints. Their identification as multi-trait heat-tolerant germplasm complements quantitative trait locus (QTL)-based approaches using interspecific populations (Cruz et al., 2023; Tene et al., 2025) by providing phenotypically validated intraspecific diversity within *P. vulgaris*, which is more immediately applicable in varietal improvement programmes. Molecular characterisation of these accessions would facilitate the identification of genomic regions underpinning their reproductive resilience and enable marker-assisted introgression into locally adapted backgrounds.

### 4.6 Night temperature, experimental context, and scope

The heat stress regime implemented in this study, characterised by a daytime temperature of 32 °C and a nighttime temperature of 22 °C, subjected plants to elevated daytime temperatures and moderately warm nights concurrently. Night temperatures exceeding 20 °C have been documented to impair pollen viability and pod set in common bean, primarily through increased respiratory carbon loss and diminished carbohydrate availability for reproductive development (Porch and Jahn, 2001; Prasad et al., 2002). Similar mechanisms have been observed in other crops, where elevated nighttime temperatures enhance maintenance respiration and deplete non-structural carbohydrate reserves, thereby constraining sink activity and yield (Aneja et al., 2022). This study does not isolate the independent effects of daytime and nighttime temperatures on reproductive and yield responses; however, the combined regime resulted in clear and consistent differentiation amongst accessions across both years. These findings are applicable to field conditions where both components of the thermal environment are elevated simultaneously.

Whilst the controlled growth chamber setting facilitates precise and reproducible temperature management, it fails to encompass the full complexity of field heat stress environments. Therefore, conducting multi-location field evaluations of the identified tolerant accessions is recommended as a subsequent step towards their integration into breeding programmes.

## 5 Conclusion

This study establishes that heat tolerance in common bean is predominantly influenced by the ability to preserve reproductive integrity, specifically pollen viability, pollen germination, stigma receptivity, and ovule viability, under elevated temperatures during the critical flowering-to-podding stage. These reproductive traits, along with seed weight components, exhibited the highest heritability estimates (60–93%) and the most consistent correlations with heat-tolerance residuals across two independent growing seasons, thereby affirming their utility as selection criteria for breeding programs. Accessions IC-14912, EC-106613, IC-545783, and IC-16906 were identified as priority donor parents for heat tolerance owing to their consistent multi-trait superiority, whereas IC-545792, EC-100098, EC-121013, and IC-362075 were characterised as heat-sensitive references. Field validation and molecular characterisation of the identified tolerant accessions are recommended as essential subsequent steps in this study.

## Data Availability

The original contributions presented in the study are included in the article/**Supplementary Material**. Further inquiries can be directed to the corresponding author.
