# Supplementary material for: Multi-trait phenotyping under reproductive-stage heat stress reveals heritable reproductive and yield indicators of tolerance in common bean (Phaseolus vulgaris L.)
Source: Front Plant Sci. 2026 Aug 11;17:1904240. doi: 10.3389/fpls.2026.1904240 (PMC13503261; doi:10.3389/fpls.2026.1904240)
Supplement: Supplementary file 1 [file SupplementaryFile1.docx]

**Figure S1:** Relationship between total seed weight under control conditions (Yc) and total seed weight under heat stress (Ys) for the first year (a) and the second year (b). The lower panels show the regression between the ratio of seed yield under heat stress relative to control (Ys/Yc) and the heat-tolerance residuals (R) for the first year (c) and the second year (d), where R = Ys – Ŷs and Ŷs represents the predicted seed yield under heat stress based on yield potential under control conditions.

**Figure S2:** Regression analysis showing the association between heat-tolerance residuals (Ys − Ŷs) and phenological and growth traits under heat stress: (a) days to flowering, (b) days to podding, (c) days to maturity, (d) height ratio, (e) biomass ratio, (f) harvest index, for the first year.

**Figure S3:** Regression analysis showing the association between heat-tolerance residuals (Ys − Ŷs) and phenological and growth traits under heat stress: (a) days to flowering, (b) days to podding, (c) days to maturity, (d) height ratio, (e) biomass ratio, (f) harvest index, for the second year.

**Figure S4:** Regression analysis showing the association between heat-tolerance residuals (Ys − Ŷs) and major yield-related traits under heat stress: (a) filled pods ratio, (b) single seed weight ratio, (c) total seed weight ratio, (d) seed number ratio, for the first year.

**Figure S5:** Regression analysis showing the association between heat-tolerance residuals (Ys − Ŷs) and major yield-related traits under heat stress: (a) filled pods ratio, (b) single seed weight ratio, (c) total seed weight ratio, (d) seed number ratio, for the second year.

**Figure S6:** Regression analysis showing the association between heat tolerance residuals (Ys – Ŷs) and major physiological traits under heat stress: (a) Electrolyte leakage ratio, (b) Stomatal conductance ratio, (c) SPAD ratio, (d) Photosynthetic efficiency ratio, for the first year.

**Figure S7:** Regression analysis showing the association between heat tolerance residuals (Ys – Ŷs) and major physiological traits under heat stress: (a) Electrolyte leakage ratio, (b) Stomatal conductance ratio, (c) SPAD ratio, (d) Photosynthetic efficiency ratio, for the second year.

**Figure S8:** Regression analysis showing the association between heat tolerance residuals (Ys – Ŷs) and reproductive biology-related traits under heat stress: (a) Pollen viability ratio, (b) Pollen germination ratio, (c) Ovule viability ratio, (d) Stigma receptivity ratio, for the first year.

**Figure S9:** Regression analysis showing the association between heat tolerance residuals (Ys – Ŷs) and reproductive biology-related traits under heat stress: (a) Pollen viability ratio, (b) Pollen germination ratio, (c) Ovule viability ratio, (d) Stigma receptivity ratio, for the second year.
