# Supplementary material for: Multi-trait phenotyping under reproductive-stage heat stress reveals heritable reproductive and yield indicators of tolerance in common bean (Phaseolus vulgaris L.)
Source: Front Plant Sci. 2026 Aug 11;17:1904240. doi: 10.3389/fpls.2026.1904240 (PMC13503261; doi:10.3389/fpls.2026.1904240)
Supplement: Supplementary file 2 [file SupplementaryFile2.docx]

**Supplementary Table 1: Two-way ANOVA for the effects of accession (A), treatment (T), replication (R), and their interaction (A*T) on 17 traits measured during the reproductive stage of 56 common bean accessions across two screening years (2023 and 2024) under control and heat stress conditions.**

| **Traits** | **Screening year** | **Accessions** | **Treatment** | **Replicates** | **Accession*Treatment** | **Error** |
| --- | --- | --- | --- | --- | --- | --- |
| **Df** |  | **55** | **1** | **2** | **55** | **222** |
| **Phenological traits** | | | | | | |
| **DP** | 1^st^ | 47.4*** | 795.5*** | 287*** | 5.7* | 4 |
|  | 2^nd^ | 62.5*** | 671.5*** | 816.7*** | 1.4** | 0.8 |
| **DM** | 1^st^ | 64.9*** | 1669.6*** | 319.3*** | 1.6* | 1 |
|  | 2^nd^ | 77.2*** | 2268.2*** | 2720.7*** | 1.7*** | 0.3 |
| **Growth traits** | | | | | | |
| **HI** | 1^st^ | 697*** | 18112*** | 382*** | 507** | 2 |
|  | 2^nd^ | 398*** | 18913*** | 8* | 77*** | 2 |
| **H** | 1^st^ | 3849*** | 6903*** | 3030*** | 22 | 185 |
|  | 2^nd^ | 3879*** | 11029*** | 4107*** | 8 | 187 |
| **BM** | 1^st^ | 88*** | 8211*** | 9*** | 30*** | 1 |
|  | 2^nd^ | 126*** | 8724*** | 7** | 16*** | 1 |
| **Yield traits** | | | | | | |
| **FP** | 1^st^ | 19.8*** | 1324.1*** | 2.3 | 0.8 | 1.6 |
|  | 2^nd^ | 27.6*** | 1669.6*** | 3.3 | 1.3 | 2.4 |
| **NS** | 1^st^ | 162*** | 17734*** | 13 | 30*** | 8 |
|  | 2^nd^ | 221*** | 22754*** | 16 | 40*** | 10 |
| **SSW** | 1^st^ | 0.0341*** | 0.5079*** | 0.0930*** | 0.0063*** | 0.0035 |
|  | 2^nd^ | 0.0402*** | 0.6599*** | 0.1223*** | 0.0078** | 0.0046 |
| **TSW** | 1^st^ | 59.6*** | 3046.0*** | 19.8*** | 13.4*** | 5 |
|  | 2^nd^ | 67*** | 3586*** | 106*** | 13*** | 4 |
| **Physiological traits** | | | | | | |
| **EL** | 1^st^ | 188*** | 8892*** | 11 | 73*** | 28 |
|  | 2^nd^ | 153*** | 26254*** | 441*** | 94*** | 38 |
| **SC** | 1^st^ | 1529*** | 57501*** | 870 | 366 | 630 |
|  | 2^nd^ | 778*** | 51050*** | 1099* | 300 | 333 |
| **SPAD** | 1^st^ | 34.4*** | 2887.6*** | 44.2 | 12.0 | 17.2 |
|  | 2^nd^ | 22* | 6040*** | 162*** | 15 | 12 |
| **PE** | 1^st^ | 0.0185*** | 0.9895*** | 0.0001 | 0.0075*** | 0.0009 |
|  | 2^nd^ | 0.0253*** | 1.1453*** | 0.0024*** | 0.0142*** | 0.0001 |
| **Reproductive traits** | | | | | | |
| **PV** | 1^st^ | 136*** | 250389*** | 192*** | 50*** | 23 |
|  | 2^nd^ | 168*** | 315778*** | 111* | 66*** | 28 |
| **PG** | 1^st^ | 168*** | 269243*** | 102 | 65*** | 35 |
|  | 2^nd^ | 140*** | 371153*** | 95* | 69*** | 29 |
| **SR** | 1^st^ | 3.4*** | 236*** | 2.8* | 1.4*** | 0.6 |
|  | 2^nd^ | 4.2*** | 286.6*** | 4.3** | 1.4** | 0.8 |
| **OV** | 1^st^ | 3.8*** | 52.1*** | 4.2*** | 0.9*** | 0.3 |
|  | 2^nd^ | 5.1*** | 171.2*** | 8.9*** | 0.9* | 0.6 |

Traits included: phenological: days to podding (DP; days) and days to maturity (DM; days); growth: harvest index (HI; %), plant height (H; cm), and biomass (BM; g plant⁻¹); yield: filled pods (FP; number plant⁻¹), number of seeds (NS; number plant⁻¹), single seed weight (SSW; g), and total seed weight (TSW; g plant⁻¹); physiological: electrolyte leakage (EL; %), stomatal conductance (SC; mmol m⁻² s⁻¹), SPAD chlorophyll index (SPAD units), and photosynthetic efficiency (PE; Fv/Fm ratio); and reproductive: pollen viability (PV; %), pollen germination (PG; %), stigma receptivity (SR; scale 1–5), and ovule viability (OV; scale 1–5).

**Supplementary Table S2. Eigenvalues, percentage of variance explained, and cumulative variance of the principal components obtained from principal component analysis (PCA) of the measured traits under heat stress during first and second year.**

| **Principal Component** | **Year 1 Eigenvalue** | **Year 1 Variance (%)** | **Year 1 Cumulative (%)** | **Year 2 Eigenvalue** | **Year 2 Variance (%)** | **Year 2 Cumulative (%)** |
| --- | --- | --- | --- | --- | --- | --- |
| **PC1** | 9.886 | 58.15 | 58.15 | 9.814 | 57.73 | 57.73 |
| **PC2** | 2.492 | 14.66 | 72.81 | 2.422 | 14.25 | 71.98 |
| **PC3** | 1.052 | 6.19 | 79.00 | 1.087 | 6.39 | 78.37 |
| **PC4** | 0.958 | 5.64 | 84.64 | 0.871 | 5.12 | 83.49 |
| **PC5** | 0.667 | 3.93 | 88.56 | 0.777 | 4.57 | 88.06 |
| **PC6** | 0.500 | 2.94 | 91.50 | 0.661 | 3.89 | 91.95 |
| **PC7** | 0.451 | 2.65 | 94.15 | 0.451 | 2.65 | 94.60 |
| **PC8** | 0.414 | 2.44 | 96.59 | 0.362 | 2.13 | 96.73 |
| **PC9** | 0.213 | 1.25 | 97.84 | 0.252 | 1.48 | 98.21 |
| **PC10** | 0.173 | 1.02 | 98.86 | 0.177 | 1.04 | 99.25 |
| **PC11** | 0.107 | 0.63 | 99.49 | 0.099 | 0.58 | 99.84 |
| **PC12** | 0.068 | 0.40 | 99.90 | 0.018 | 0.10 | 99.94 |
| **PC13** | 0.010 | 0.06 | 99.96 | 0.005 | 0.03 | 99.97 |
| **PC14** | 0.004 | 0.02 | 99.98 | 0.003 | 0.02 | 99.99 |
| **PC15** | 0.002 | 0.01 | 99.99 | 0.002 | 0.01 | 100.00 |
| **PC16** | 0.002 | 0.01 | 100.00 | 0.001 | 0.00 | 100.00 |
| **PC17** | 0.000 | 0.00 | 100.00 | 0.000 | 0.00 | 100.00 |

**Supplementary Table S3. Variable loadings of all measured traits on principal components (PC1–PC17) under heat stress during the first and second year.**

| **Traits** | **Year** | **PC1** | **PC2** | **PC3** | **PC4** | **PC5** | **PC6** | **PC7** | **PC8** | **PC9** | **PC10** | **PC11** | **PC12** | **PC13** | **PC14** | **PC15** | **PC16** | **PC17** |
| --- | --- | --- | --- | --- | --- | --- | --- | --- | --- | --- | --- | --- | --- | --- | --- | --- | --- | --- |
| SPAD | Year 1 | 0.589 | 0.376 | 0.259 | -0.344 | 0.159 | -0.237 | 0.472 | 0.125 | -0.065 | 0.034 | -0.025 | 0.018 | 0.001 | 0 | 0 | -0.001 | 0 |
|  | Year 2 | 0.492 | -0.327 | -0.037 | 0.382 | -0.501 | 0.468 | -0.022 | -0.171 | 0.002 | 0.056 | -0.027 | -0.003 | 0 | 0 | -0.001 | 0 | 0 |
| SC | Year 1 | 0.693 | 0.128 | -0.325 | -0.355 | 0.088 | 0.378 | 0.181 | -0.199 | 0.217 | -0.026 | 0.016 | -0.014 | 0.012 | 0.001 | 0.001 | 0 | 0 |
|  | Year 2 | 0.699 | -0.123 | -0.314 | -0.213 | -0.248 | -0.386 | 0.029 | -0.266 | 0.25 | 0.083 | 0.028 | 0.005 | -0.001 | 0.001 | 0 | 0 | 0 |
| FP | Year 1 | 0.831 | 0.375 | 0.125 | -0.114 | -0.042 | -0.048 | -0.165 | -0.009 | 0.027 | 0.268 | 0.176 | -0.069 | -0.004 | -0.002 | -0.001 | 0 | 0 |
|  | Year 2 | 0.833 | -0.347 | 0.113 | 0.041 | -0.065 | -0.041 | 0.193 | -0.067 | -0.015 | -0.333 | 0.114 | 0.002 | 0.003 | 0 | 0.001 | 0 | 0 |
| NS | Year 1 | 0.857 | 0.382 | 0.17 | 0.268 | -0.101 | 0.008 | -0.008 | -0.04 | 0.037 | -0.023 | -0.012 | 0.059 | 0.015 | -0.011 | 0.031 | 0.011 | 0 |
|  | Year 2 | 0.862 | -0.373 | 0.216 | 0.126 | 0.215 | -0.033 | -0.025 | 0.004 | 0.033 | 0.064 | -0.019 | 0.002 | 0.014 | 0.026 | 0.03 | 0 | 0 |
| PE | Year 1 | 0.757 | 0.176 | -0.116 | -0.126 | 0.073 | 0.269 | -0.154 | 0.506 | -0.049 | 0.008 | -0.078 | -0.019 | 0.004 | 0.001 | 0.001 | 0 | 0 |
|  | Year 2 | 0.752 | 0.032 | -0.069 | 0.131 | -0.377 | -0.147 | -0.001 | 0.487 | 0.094 | 0.017 | 0.025 | 0.008 | -0.001 | 0 | 0.001 | 0 | 0 |
| EL | Year 1 | -0.589 | -0.193 | -0.351 | 0.566 | -0.019 | 0.09 | 0.329 | 0.163 | 0.048 | 0.158 | 0.048 | 0.007 | 0.001 | 0.002 | 0 | 0.001 | 0 |
|  | Year 2 | -0.474 | 0.225 | -0.479 | 0.556 | 0.355 | 0.079 | 0.074 | 0.015 | 0.209 | -0.031 | 0.049 | 0.004 | -0.001 | 0 | 0 | 0 | 0 |
| PV | Year 1 | 0.869 | 0.363 | 0.16 | 0.267 | -0.094 | 0.033 | -0.007 | -0.027 | 0.043 | -0.029 | -0.018 | 0.052 | 0.018 | 0.01 | -0.015 | -0.004 | 0 |
|  | Year 2 | 0.86 | -0.383 | 0.229 | 0.11 | 0.204 | -0.034 | -0.013 | 0.006 | 0.04 | 0.061 | -0.019 | 0.025 | 0.012 | -0.012 | -0.014 | 0 | 0 |
| PG | Year 1 | 0.869 | 0.363 | 0.16 | 0.267 | -0.094 | 0.033 | -0.007 | -0.027 | 0.043 | -0.029 | -0.018 | 0.052 | 0.018 | 0.01 | -0.015 | -0.004 | 0 |
|  | Year 2 | 0.86 | -0.383 | 0.229 | 0.11 | 0.204 | -0.034 | -0.013 | 0.006 | 0.04 | 0.061 | -0.019 | 0.025 | 0.012 | -0.012 | -0.014 | 0 | 0 |
| H | Year 1 | 0.089 | 0.616 | -0.42 | 0.191 | 0.589 | -0.157 | -0.125 | -0.096 | -0.046 | -0.006 | -0.021 | 0.004 | -0.001 | 0 | 0 | 0 | 0 |
|  | Year 2 | 0 | -0.621 | -0.314 | -0.524 | 0.149 | 0.423 | 0.024 | 0.133 | 0.145 | -0.012 | 0.006 | 0.001 | 0 | 0 | 0 | 0 | 0 |
| DP | Year 1 | -0.746 | -0.009 | 0.453 | 0.078 | 0.344 | 0.232 | 0.018 | 0.096 | 0.008 | -0.122 | 0.184 | 0.046 | -0.004 | -0.003 | 0 | -0.001 | 0 |
|  | Year 2 | -0.793 | 0.166 | 0.438 | -0.01 | -0.087 | 0.023 | 0.166 | 0.007 | 0.272 | -0.1 | -0.177 | -0.011 | 0.004 | -0.001 | 0.001 | 0 | 0 |
| DM | Year 1 | -0.777 | -0.005 | 0.452 | 0.046 | 0.214 | 0.226 | -0.024 | -0.138 | 0.012 | 0.208 | -0.172 | -0.001 | -0.001 | 0 | 0 | 0.001 | 0 |
|  | Year 2 | -0.789 | 0.118 | 0.489 | -0.045 | -0.038 | 0.121 | 0.171 | -0.003 | 0.11 | 0.143 | 0.212 | -0.002 | -0.004 | 0.001 | 0 | 0 | 0 |
| BM | Year 1 | 0.85 | -0.107 | -0.068 | 0.088 | -0.035 | 0.263 | 0.102 | -0.187 | -0.368 | -0.007 | 0.028 | -0.031 | -0.003 | -0.001 | 0 | 0 | 0 |
|  | Year 2 | 0.831 | 0.09 | -0.099 | -0.011 | 0.086 | 0.018 | 0.508 | 0.012 | -0.105 | 0.091 | -0.064 | 0.008 | -0.034 | 0.001 | 0 | 0 | 0 |
| HI | Year 1 | 0.833 | -0.269 | 0.238 | 0.301 | 0.135 | -0.057 | 0.069 | 0.008 | 0.091 | -0.102 | -0.032 | -0.202 | -0.004 | -0.003 | 0.001 | -0.001 | 0 |
|  | Year 2 | 0.88 | 0.226 | 0.217 | -0.006 | 0.112 | 0.086 | -0.3 | -0.022 | 0.107 | -0.052 | 0.008 | 0.009 | -0.057 | 0.002 | 0 | 0 | 0 |
| TSW | Year 1 | 0.977 | -0.15 | 0.004 | 0.077 | 0.017 | 0.028 | 0.004 | -0.01 | 0.062 | 0.006 | -0.022 | 0.065 | -0.091 | -0.009 | -0.002 | -0.001 | 0 |
|  | Year 2 | 0.978 | 0.124 | 0.032 | 0.007 | 0.101 | 0.019 | -0.001 | 0.016 | 0.024 | 0.03 | 0.011 | -0.12 | 0.006 | -0.008 | -0.003 | 0 | 0 |
| SSW | Year 1 | 0.738 | -0.647 | 0.017 | -0.038 | 0.163 | -0.064 | -0.031 | -0.001 | 0.007 | 0.028 | 0.014 | 0.033 | 0.01 | 0.022 | -0.007 | 0.027 | 0 |
|  | Year 2 | 0.765 | 0.619 | -0.01 | -0.103 | 0.02 | 0.135 | 0.011 | -0.014 | 0.017 | -0.02 | 0.003 | 0.009 | 0.018 | 0.035 | -0.023 | 0 | 0 |
| SR | Year 1 | 0.743 | -0.641 | 0.017 | -0.019 | 0.155 | -0.063 | -0.048 | -0.009 | 0.009 | 0.044 | 0.01 | 0.048 | 0.009 | 0.03 | 0.015 | -0.021 | 0 |
|  | Year 2 | 0.749 | 0.637 | -0.007 | -0.109 | 0.004 | 0.14 | 0.008 | -0.025 | 0.018 | -0.004 | 0.008 | 0.022 | 0.015 | -0.016 | 0.012 | 0 | 0 |
| OV | Year 1 | 0.755 | -0.632 | -0.022 | -0.002 | 0.132 | -0.058 | -0.026 | 0.011 | 0.012 | 0.045 | -0.002 | 0.053 | 0.033 | -0.047 | -0.007 | -0.005 | 0 |
|  | Year 2 | 0.749 | 0.637 | -0.007 | -0.109 | 0.004 | 0.14 | 0.008 | -0.025 | 0.018 | -0.004 | 0.008 | 0.022 | 0.015 | -0.016 | 0.012 | 0 | 0 |

Traits included: phenological: days to podding (DP; days) and days to maturity (DM; days); growth: harvest index (HI; %), plant height (H; cm), and biomass (BM; g plant⁻¹); yield: filled pods (FP; number plant⁻¹), number of seeds (NS; number plant⁻¹), single seed weight (SSW; g), and total seed weight (TSW; g plant⁻¹); physiological: electrolyte leakage (EL; %), stomatal conductance (SC; mmol m⁻² s⁻¹), SPAD chlorophyll index (SPAD units), and photosynthetic efficiency (PE; Fv/Fm ratio); and reproductive: pollen viability (PV; %), pollen germination (PG; %), stigma receptivity (SR; scale 1–5), and ovule viability (OV; scale 1–5).
